# Microbial Communities of Deep-Sea Methane Seeps at Hikurangi Continental Margin (New Zealand)

**DOI:** 10.1371/journal.pone.0072627

**Published:** 2013-09-30

**Authors:** S. Emil Ruff, Julia Arnds, Katrin Knittel, Rudolf Amann, Gunter Wegener, Alban Ramette, Antje Boetius

**Affiliations:** 1 HGF MPG Group for Deep Sea Ecology and Technology, Max Planck Institute for Marine Microbiology, Bremen, Germany; 2 Department of Molecular Ecology, Max Planck Institute for Marine Microbiology, Bremen, Germany; 3 MARUM Center for Marine Environmental Sciences, University of Bremen, Bremen, Germany; 4 Alfred Wegener Institute Helmholtz Center for Polar and Marine Research, Bremerhaven, Germany; Royal Netherlands Institute of Sea Research (NIOZ), Netherlands

## Abstract

The methane-emitting cold seeps of Hikurangi margin (New Zealand) are among the few deep-sea chemosynthetic ecosystems of the Southern Hemisphere known to date. Here we compared the biogeochemistry and microbial communities of a variety of Hikurangi cold seep ecosystems. These included highly reduced seep habitats dominated by bacterial mats, partially oxidized habitats populated by heterotrophic ampharetid polychaetes and deeply oxidized habitats dominated by chemosynthetic frenulate tubeworms. The ampharetid habitats were characterized by a thick oxic sediment layer that hosted a diverse and biomass-rich community of aerobic methanotrophic *Gammaproteobacteria*. These bacteria consumed up to 25% of the emanating methane and clustered within three deep-branching groups named Marine Methylotrophic Group (MMG) 1-3. MMG1 and MMG2 methylotrophs belong to the order *Methylococcales*, whereas MMG3 methylotrophs are related to the *Methylophaga* . Organisms of the groups MMG1 and MMG3 are close relatives of chemosynthetic endosymbionts of marine invertebrates. The anoxic sediment layers of all investigated seeps were dominated by anaerobic methanotrophic archaea (ANME) of the ANME-2 clade and sulfate-reducing *Deltaproteobacteria*. Microbial community analysis using Automated Ribosomal Intergenic Spacer Analysis (ARISA) showed that the different seep habitats hosted distinct microbial communities, which were strongly influenced by the seep-associated fauna and the geographic location. Despite outstanding features of Hikurangi seep communities, the organisms responsible for key ecosystem functions were similar to those found at seeps worldwide. This suggests that similar types of biogeochemical settings select for similar community composition regardless of geographic distance. Because ampharetid polychaetes are widespread at cold seeps the role of aerobic methanotrophy may have been underestimated in seafloor methane budgets.

## Introduction

Hikurangi margin located off northeastern New Zealand is an accretionary margin originating from the subduction of the Pacific plate under the Australian plate. The subduction causes dewatering of Hikurangi trough sediments and forms fluids enriched in hydrocarbons that migrate through the deformation front [1]. Some of the gas transported with the fluids forms gas hydrates in the seabed, but a substantial proportion is emitted to the water column [2,3,4,5]. Hikurangi margin was suggested to be a biogeographically new cold seep province, since many of the local seeps were populated by novel animal species, and the communities showed a high degree of endemism [6]. A new type of seep habitat was described dominated by heterotrophic ampharetids (*Ampharetidae*, *Polychaeta*) [7], with isotopic signatures revealing a methane-based nutrition of the worms [8]. Ampharetid seep habitats are biogeochemically distinct due to their high methane effluxes [2] and high total oxygen uptake (TOU) rates, indicating that aerobic methanotrophy is a major pathway for methane turnover [7]. Furthermore, it was suggested that ampharetid habitats represent an early stage in the development of seep ecosystems [7]. Hikurangi margin also hosts highly reduced seep ecosystems characterized by mats of sulfur-oxidizing bacteria that scavenge sulfur released by the anaerobic oxidation of methane (AOM) [9]. A third type of seep habitat is inhabited by frenulate tubeworms (*Siboglinidae*, *Polychaeta*), which root in subsurface sediments and harbor bacterial endosymbionts [6]. It was shown that these different types of dominating megafauna indicate spatial variations in fluid flow, methane turnover and efflux to the hydrosphere [10,11,12].

In most previously studied methane seeps the biogeochemistry was dominated by AOM in subsurface horizons and sulfide oxidation at the surface. AOM is mediated by consortia of anaerobic methanotrophic archaea (ANME) and sulfate-reducing bacteria (SRB). ANME belong to the class *Methanomicrobia* and comprise the major clades ANME-1, ANME-2 and ANME-3 [9]. The partner SRB are usually *Deltaproteobacteria* closely related to *Desulfosarcina* */Desulfococcus* (DSS) and *Desulfobulbus* (DBB) [9,13]. It is generally assumed that aerobic methanotrophy plays a minor role at seeps due to limited oxygen availability in the seafloor [9]. Exceptions are the centers of active mud volcanoes, where high pore water velocities prevent the diffusion of sulfate into the sediment and hence inhibit AOM [10,14], or where other disturbances favor the more rapidly growing aerobic methanotrophs [11]. Typical aerobic methanotrophs at seeps are *Gammaproteobacteria* of the order *Methylococcales* [14,15,16].

A main objective of this study was to compare the microbial communities of the different Hikurangi cold seeps, with a focus on ampharetid habitats, to identify distinct distribution patterns. Given the large geographic distances to other known seep ecosystems, and the previous description of a distinct seep fauna at the Hikurangi margin, another objective of this study was to compare the microbial communities of the Hikurangi region with other seeps worldwide. A detailed description of the new seep habitats is provided based on biogeochemical measurements, comparative sequence analyses of 16S rRNA and the particulate methane monooxygenase subunit A (*pmoA*), and visualization of key methanotrophs using fluorescence in situ hybridization (FISH). Microbial community β-diversity at Hikurangi cold seeps was analyzed by the fingerprinting technique ARISA [17]. We showed that aerobic methanotrophy plays an important role at ampharetid habitats and found distinct microbial communities that differ from other types of seep and non-seep ecosystems.

## Materials and Methods

### Sampling procedure and study sites

**Figure 1:**
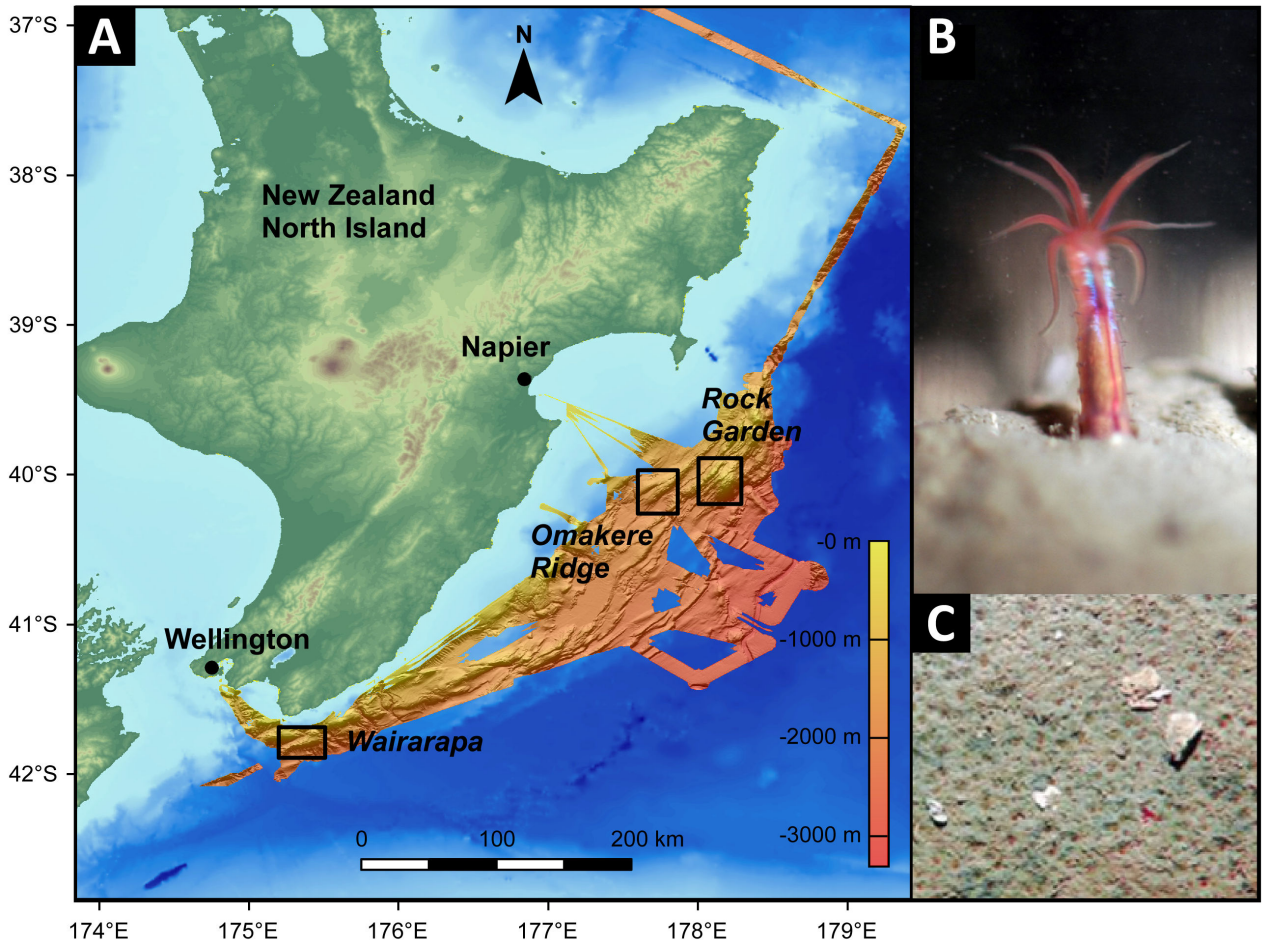
The sampling sites. **A**: Bathymetric map of Hikurangi margin, New Zealand. The map was generated using the ESRI ArcGIS software and the General Bathymetric Chart of the Oceans (GEBCO_09 Grid, version 20091120, http://www.gebco.net). Data for the high resolution bathymetry were provided by Jens Greinert. Sampling areas are indicated. **B**: Close-up view of an ampharetid polychaete, the dominant fauna at many Hikurangi seep sites. These polychaetes are between 1 and 2.5 cm long and inhabit tubes which extend 2-3 cm into the sediment (image courtesy of S. Sommer). **C**: Each depression on the sediment surface was created and inhabited by a single polychaete (image courtesy of D. Bowden).

The nine investigated cold seep sites were located on the Hikurangi continental margin, east of New Zealand’s North Island (Table 1, Figure 1). Seven sites were dominated by heterotrophic ampharetids, one site showed few ampharetids and a white surface, supposedly caused by mat-forming sulfur-oxidizing bacteria (SOB), and one site was densely populated by frenulate tubeworms. The sites 45, 78 and 232 were located at Omakere Ridge and the sites 215 and 258 were located at Rock Garden, on the northern part of Hikurangi margin (Figure 1, Table 1). The sites 124, 157, 273, 309 and 315 were situated at Wairarapa several hundred kilometers further south on Hikurangi margin. The samples were retrieved by TV-guided multicoring during cruise SO-191 leg 2 and 3 on the German research vessel “FS Sonne” in 2007. After retrieval, the cores were transferred to the cold room (4°C) and sectioned at intervals of 1 or 2 cm. Five representative sites were selected for a detailed microbial community analysis, These were site 45 (frenulate habitat), 124, 309 (ampharetid habitat), 315 (SOB habitat) and 78 (reference site). Subsamples for DNA extraction were frozen at -20°C and subsamples for FISH were fixed in 4% formaldehyde for 24 h at 4 °C, washed twice with 1× PBS and stored in 1× PBS/absolute ethanol (1:1) at -20 °C. As a formal process in the preparation of the FS SONNE expedition 191, a diplomatic permission was received from the coastal state (New Zealand) to retrieve samples for scientific purposes from their Exclusive Economic Zone (EEZ, 200 miles offshore). No protected species were harmed by the seafloor sampling activities.

**Table 1:** Sampling sites included in this study (FS Sonne, cruise SO 191 – New Vents; 11.01. - 23.03.2007).

**Station No.**	**PANGAEA Event**	**Area**	**Habitat**	**Latitude [S]**	**Longitude [E]**	**Water depth [m]**
45	SO191/2_045	Omakere Ridge	*Frenulata*	40°01.079'	177°51.573'	1159
78	SO191/2_078	Omakere Ridge	Reference	40°01.399'	177°48.944'	1182
124	SO191/2_124	Wairarapa N. Tower	*Ampharetidae*	41°46.908'	175°24.024'	1054
157	SO191/2_157	Wairarapa N. Tower	*Ampharetidae*	41°46.851'	175°24.107'	1056
215	SO191/3_215	Rock Garden	*Ampharetidae*	40°02.007'	178°09.650'	661
232	SO191/3_232	Omakere Ridge	*Ampharetidae*	40°02.150'	177°47.950'	1172
258	SO191/3_258	Rock Garden	*Ampharetidae*	40°01.890'	178°09.650'	659
273	SO191/3_273	Wairarapa	*Ampharetidae*	41°46.986'	175°24.251'	1059
309	SO191/3_309	Wairarapa Takahae	*Ampharetidae*	41°46.350'	175°25.690'	1057
315	SO191/3_315	Wairarapa Takahae	SOB	41°46.320'	175°25.690'	1057

### Concentration of methane and sulfur species and biogeochemical rate measurements

For methane measurements, 2 ml sediment of each horizon was sampled with cut-off plastic syringes immediately after sectioning. Material was transferred into 20 ml butyl-rubber sealed vials filled with 5 ml sodium hydroxide solution (2.5%). From the headspace of these vials methane concentrations were measured using gas chromatography coupled to flame-ionization detection (GC 5890A, Hewlett Packard, Palo Alto, CA, USA). Sulfide was determined colorimetrically from pore water samples fixed with zinc acetate (20%) using methylene blue method [18]. Sulfate concentrations were determined by ion chromatography (761 Compact IC, Ω Metrohm, Filderstadt, Germany).

Methane oxidation (MOx) and sulfate reduction (SR) rates were determined using the subcore injection method [19]. Three subcores from the sampling sites were treated with carrier-free radiolabeled methane (5 kBq) or sulfate (50 kBq), respectively. Cores were incubated at 4°C for 12 to 36 hours in the dark. For MOx measurements biological activity was stopped by the transfer of 1 cm long sediment sections into gas tight flasks with sodium hydroxide solution (2.5%). To determine MOx rates, concentration of the reactant methane was determined by flame ionization gas chromatography (GC 5890A), ^14^C-methane was stripped from the headspace, converted to CO_2_ (via CuO-catalyzed oxidation at 850°C) and trapped in CarboSorb (PerkinElmer, Waltham, MA, USA). Microbially produced inorganic carbon was released from the sample by acidification and trapping in CarboSorb. To determine SR rates, sediment sections were transferred to zinc acetate solution. Concentration of sulfate was measured via ion chromatography or by barium sulfate precipitation. Activity of added ^35^S-sulfate was measured from the supernatant, activity of produced total reduced sulfur was determined after releasing it using cold chromium distillation [20]. To determine activities of ^14^C and ^35^S, scintillation cocktails Permafluor-E© or Ultima Gold© (PerkinElmer) were added and samples measured by scintillation technique (Tricarb 2500 liquid scintillation counter, Packard, Palo Alto, CA, USA). Rates were determined by the ratio of product and reactant activity and the reactant concentration as described before [21].

### DNA extraction

DNA was retrieved from 3 g of sediment (pooled for each 5 cm layer) by chloroform extraction as described before [22] and purified using the Wizard DNA clean-up system (Promega, Madison, WI, USA).

### Gene library construction

PCRs for 16S rRNA gene libraries were carried out using 5-75 ng environmental DNA, 25 cycles and the primers GM3/GM4 and Arch20F/Uni1392R (for details see Table S2 in the Materials and Methods S1). 10 parallel PCRs of each sample were pooled, purified using the PCR Purification Kit (Qiagen, Hilden, Germany) and eluted in 30 µl ultrapure water. For the bacterial library of station 315 we used 15 cycles and pooled 20 PCRs. Cloning reactions were performed with vector pCR4 and the TOPO TA Cloning Kit (Invitrogen, San Diego, CA, USA) and inserts sequenced using the BigDye Terminator v 3.1 Cycle Sequencing Kit (Applied Biosystems, Carlsbad, CA, USA) on an ABI PRISM 3130xl Genetic Analyzer. Bacterial libraries of station 45 and 78 and archaeal libraries of station 78 and 315 were constructed alike, except that vector pGEM T-Easy (Promega, Madison, WI, USA) was used and sequencing was performed by LGC Genomics (LGC, Berlin, Germany). The libraries for the particulate methane monooxygenase subunit A gene (*pmoA*) were constructed using 24 cycles and the primers A189F/MB661R (for details see Table S2 in the Materials and Methods S1). Chimeric 16S rRNA sequences were removed using the software Mallard [23].

### Phylogenetic analysis

Phylogenetic classification was carried out with the software package ARB [24] based on the SILVA small subunit rRNA reference sequence database (SSURef v102, release date: 02-15-10) [25]. Sequences were aligned by SINA [26] and manually optimized according to the secondary structure. To build phylogenetic trees our full-length sequences (>1300 bases) were complemented with sequences from the database. Phylogenetic trees were calculated with the maximum likelihood algorithm PHYML (100 bootstraps) as implemented in ARB using a positional variability filter. Nucleotide substitutions were weighted according to the HKY model [27]. Partial sequences were added to the tree using the ARB maximum parsimony algorithm. Redundant sequences were deleted for clarity.

### Clustering of operational taxonomic units (OTUs) and determination of species richness

A lower triangular distance matrix of the sequences of each gene library was calculated using the neighbor-joining method [28] as implemented in ARB. The 16S and *pmoA* matrices were corrected according to Jukes-Cantor [29] and Kimura [30], respectively. OTU clustering, species rarefaction and Chao1 OTU richness estimates [31] were performed based on these matrices using the software mothur v 1.20 [32].

### Automated ribosomal intergenic spacer analysis (ARISA)

ARISA was carried out using 20-25 ng of environmental DNA according to Böer and colleagues [33], with slight modifications (see Materials and Methods S1). To include the maximum number of peaks while excluding background fluorescence, only fragments above a threshold of 50 fluorescence units and between 100 and 1000 bp were taken into consideration [34]. The GeneMapper output file was reformatted and analyzed by custom R scripts [35]. An “interactive” binning strategy with a bin size of 2 bp was applied to the ARISA generated data to account for size calling imprecision [34]. The binning frame that offered the highest pairwise similarities among samples was further subjected to multivariate analyses. An operational taxonomic unit (OTU) was considered present in a given DNA sample only if it was observed at least twice among the set of three replicated PCRs from the DNA extract of that particular sample [35,36]. We processed 26 samples originating from different geographic locations, seep habitats, sampling sites and depth intervals.

### Statistical analyses

Non-metric multidimensional scaling (NMDS) [37] was carried out based on the Bray-Curtis similarity measure [38] using PAST v 1.99 [39]. To reduce the stress value, a three-dimensional (3D) ordination space was chosen of which two axes are shown. Stress values below 0.2 reliably represent the underlying data. Analysis of similarity (ANOSIM) was used to determine significant differences between groups [40] based on a Bray-Curtis similarity measure. Redundancy analysis (RDA) and analysis of variance (ANOVA) was performed using the software package vegan [41] based on the software environment R v 2.15.0.

### Cell enumeration and catalyzed reporter deposition fluorescence in situ hybridization (CARD–FISH)

Total numbers of single cells were determined using acridine orange direct counts [11]. CARD-FISH was carried out as described previously [42] with the following modifications. The samples were ultra-sonicated before filtration and endogenous peroxidases inactivated by incubation in 0.15% H_2_O_2_ in methanol for 30 min at room temperature [43]. Archaeal cell walls were permeabilized with Proteinase K solution (15 µg ml^-1^ (Merck, Darmstadt, Germany) in 0.05 M EDTA (pH 8), 0.1 M Tris-HCl (pH 8), 0.5 M NaCl) for 5 min or HCl (0.1M) for 1 min at room temperature. Bacterial cell walls were permeabilized with lysozyme solution (1000kU/ml) [42] for 30 min at 37°C or HCl (0.1M) for 1 min at room temperature. In case of dual CARD-FISH, probe-coupled peroxidases of the first hybridization were inactivated (see above) prior to the second hybridization. Cells were stained with DAPI (1µg/ml), embedded in mounting medium and counted in 20-60 independent microscopic fields using an Axiophot II epifluorescence microscope (Carl Zeiss, Jena, Germany). Cells in dense aggregates were estimated semi-quantitatively in 100-250 independent microscopic fields [14]. A complete list of probes used in this study is provided (see Table S2 in the Materials and Methods S1).

### Nucleotide sequence accession numbers

The nucleotide sequence data reported in this paper have been archived in the EMBL, GenBank, and DDBJ nucleotide sequence database. The accession numbers JF268327 to JF268425, JN884818 to JN885079 and JQ241648 to JQ241771 refer to 16S rRNA gene sequences, JN990380 to JN990410 and KC751343 to KC751411 refer to *pmoA* gene sequences. Submission was done using the new tool CDinFusion [44]. Contextual data are included according to the MIMARKS standard [45]. 

## Results

In this study we investigated nine cold seep sites located off the northeast coast of New Zealand at water depths between 660 and 1200 meters (Figure 1, Table 1). Seven of the investigated seep sites (124,157,215,232,258,273,309) were densely populated by heterotrophic ampharetids (*Ampharetidae*, *Polychaeta*). One seep site (315) hosted mainly sulfur-oxidizing bacteria (SOB) with minor co-occurrence of ampharetid polychaetes and one seep site (45) was colonized by chemosynthetic frenulate tubeworms (*Siboglinidae*, *Polychaeta*). A site without see page (78) was analyzed as reference.

### Biogeochemistry of Hikurangi seep ecosystems

**Figure 2:**
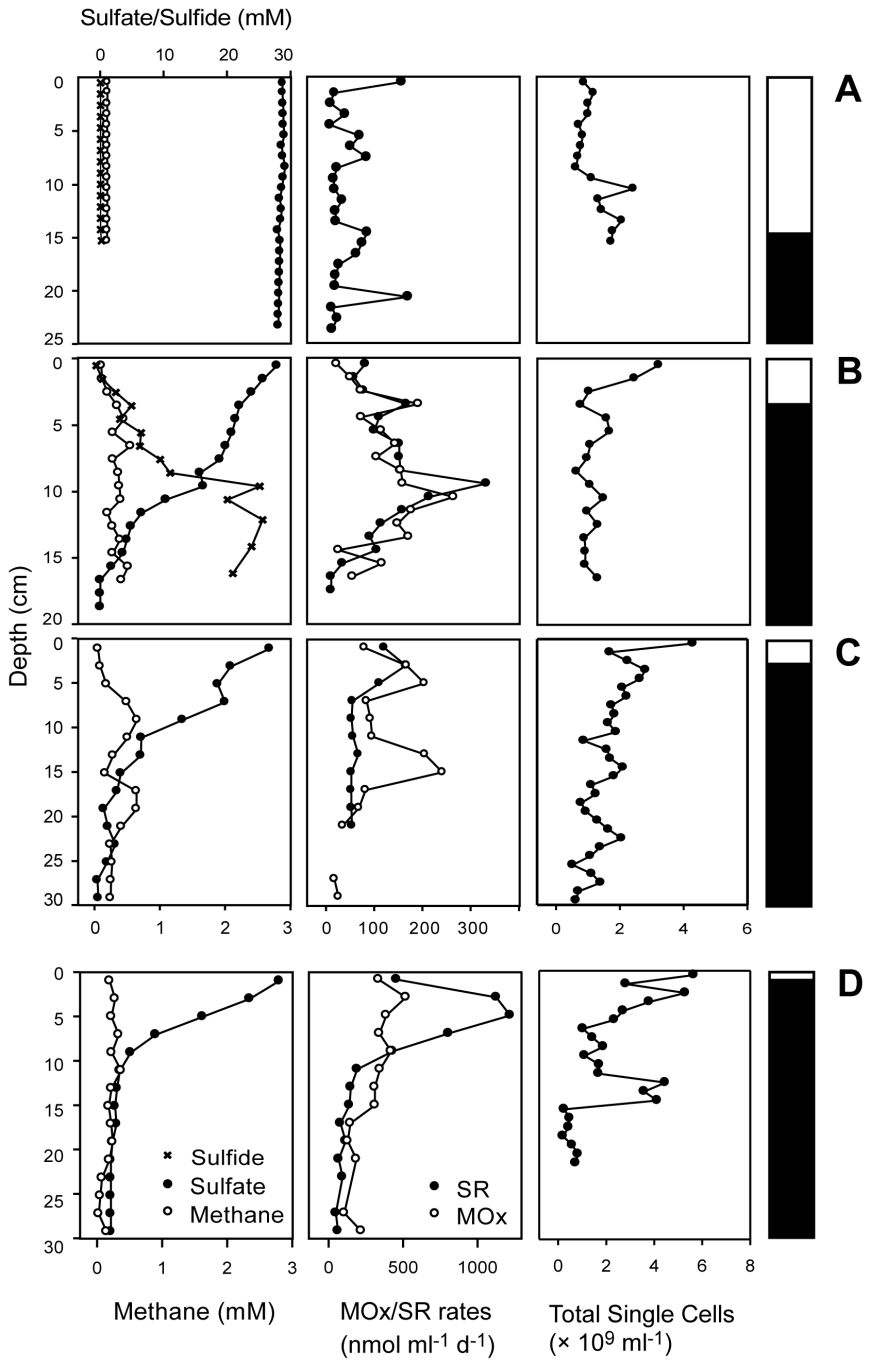
Biogeochemistry and total single cell numbers. Methane and sulfate concentrations, rates of methane oxidation (MOx) and sulfate reduction (SR), and total single cell numbers of four sampling sites from the Hikurangi margin. **A**: Frenulate site 45, **B**: Ampharetid site 124, **C**: Ampharetid site 309, D:SOB site 315. Note the different scales on x- and y-axes. The bars on the side show the redox state of the sediment: White is oxic/suboxic and black is sulfidic. In the frenulate habitat, methane hardly reached the top 10 cm layer and the suboxic zone extended to 15 cmbsf. In the ampharetid habitat, methane was present throughout the core, but the suboxic zone nevertheless reached 2-4 cm deep. In the bacterial mat habitat, the suboxic zone was limited to a few mm.

Biogeochemical measurements were conducted at all seep sites with a focus on the ampharetid habitats 124 and 309, the SOB habitat 315 and the frenulate habitat 45. We distinguished between oxic/suboxic and sulfidic sediment layers based on sulfide concentration or appearance of the sediment cores. At the frenulate site 45 methane concentrations [CH_4_] remained below 3 µM down to 16 cmbsf (centimeter below sea floor) and sulfate [SO_4_^2-^] was nearly constant at 28 mM. Methane oxidation (MOx) was not detected and sulfate reduction (SR) was present at very low rates (Figure 2). At the ampharetid sites 124 and 309, [CH_4_] reached up to 600 µM and [SO_4_^2-^] decreased from 28 mM to 0 mM within the upper 20 cmbsf. The sulfide concentration at site 124 was low in the upper sediment horizon (0-7 cm), but increased strongly below. Although sulfide data were not available for site 309, this trend was observed at this and other ampharetid sites (Figure S1), indicating that the upper sediment horizon at ampharetid sites is non-sulfidic. At both ampharetid sites the MOx activity showed a peak in the oxic and anoxic layer (Figure 2), which was also observed at most other ampharetid sites (Figure S1). Within the oxic/suboxic layer methane oxidation rates amounted to 1.2 ± 0.5 mol m^-2^ yr^-1^ (mean ± standard error) at site 124 and 1.8 ± 0.2 mol m^-2^ yr^-1^ at site 309. In the sulfidic layers integrated MOx rates were higher with 4.3 ± 1.2 mol m^-2^ yr^-1^ (site 124) and 6.1 ± 1.9 mol m^-2^ yr^-1^ (site 309). Integrated SR rates were 5.0 ± 1.9 mol m^-2^ yr^-1^ (site 124) and 2.9 ± 0.8 mol m^-2^ yr^-1^ (site 309). The average annual methane oxidation rate found in the oxic layer of all ampharetid sites was around 0.7 ± 0.2 mol m^-2^ yr^-1^, as compared to 3.7 ± 1.1 mol m^-2^ yr^-1^ in the anoxic zone.

At the SOB site 315 the oxic/suboxic layer was only a few millimeters thick. Methane concentration was up to 400 µM and sulfate disappeared within the upper 10 cmbsf (Figure 2). Sulfide data were not available for this site. MOx and SR rates peaked between 3 and 5 cmbsf. Integrated MOx rates were 14.6 ± 2.2 mol m^-2^ yr^-1^ within the upper 10 cmbsf and 9.1 ± 2.7 mol m^-2^ yr^-1^ between 10 and 20 cmbsf. Integrated SR rates were 21.4 ± 2.9 mol m^-2^ yr^-1^ and 3.7 ± 0.9 mol m^-2^ yr^-1^, respectively. At the reference site we did not detect methane, sulfide, AOM or SR (Figure S1).

### Archaeal diversity of Hikurangi margin sediments

Sequencing of selected clones from 16S rRNA gene libraries of the upper 10 cmbsf from four sampling sites (45,78,309,315) resulted in a total of 305 partial and 21 full-length, high quality sequences. We found the highest archaeal richness at the reference site with 27 observed and 48 estimated operational taxonomic units (OTUs) using Chao1 at a 97% similarity cut-off. The lowest archaeal richness was present at the SOB site (14 observed and 25 estimated OTUs). At the frenulate and ampharetid sites 16 of 28 and 18 of 46 estimated OTUs were retrieved, respectively.

**Figure 3:**
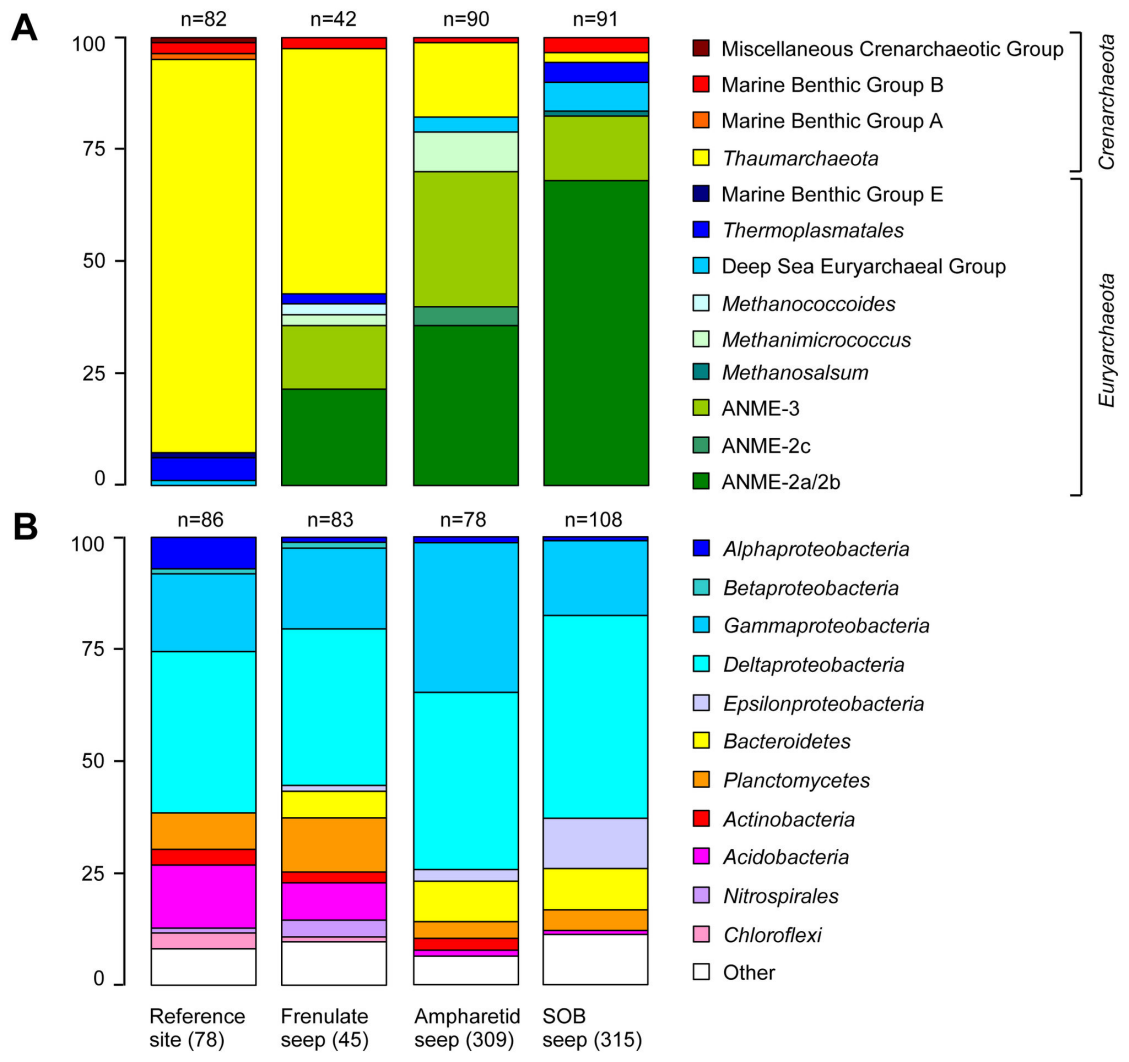
Relative 16S rRNA clone frequencies. **A**: Archaeal and B: bacterial diversity at the frenulate (45), ampharetid (309), SOB (315) and the reference site (78) as determined by 16S rRNA gene analysis. The scale bar represents relative clone frequency in percent. The total number of clones per library is indicated above the respective column.

At the reference site, we detected mostly *Thaumarchaeota* and *Crenarchaeota* of Marine Benthic Group B, whereas sequences of methane-oxidizing groups were absent (Figure 3A). Gene libraries of methane seep sites 45, 309 and 315 contained many sequences related to ANME-2a/2b and ANME-3. ANME-2c was found only at the ampharetid site 309. Interestingly, ANME-1 sequences were absent in all libraries. The diversity of ANME at each site was high, especially at site 309 with 17 different OTUs (97% cut off). A few sequences affiliated to methanogenic *Methanosarcinaceae* were present at all seep sites. A detailed archaeal phylogeny is provided (Figure S2).

### Bacterial diversity of Hikurangi margin sediments

The upper 10 cmbsf of all sampled sites (45,78,309,315) yielded a total of 355 bacterial 16S rRNA partial and 78 full-length sequences. These were distributed widely among the *Bacteria* (Figure 3B). The highest bacterial richness was found at the reference site, with 77 observed and 1355 estimated OTUs at a 97% similarity cut-off. Lowest richness was present at the SOB site (54 observed and 56 estimated OTUs). At the frenulate and ampharetid sites we retrieved 62 of 197 and 54 of 177 estimated OTUs, respectively.

The majority of sequences clustered with the *Alphaproteobacteria, Gammaproteobacteria* and *Deltaproteobacteria*, *Planctomycetes* and *Acidobacteria*, which were present in all sediments. *Deltaproteobacteria* was the dominant phylum in all libraries and the majority of the clones was assigned to sulfate reducers (Figure S3). Among those, members of the *Desulfobulbaceae*, such as SEEP-SRB3 and SEEP-SRB4 were only found at active methane seeps, whereas *Desulfobacteraceae* were found in all sediments. Within the *Desulfobacteraceae* the genus *Desulfobacula* was confined to the seep sediments, whereas members of the SEEP-SRB1 were also found at the reference site.

**Figure 4:**
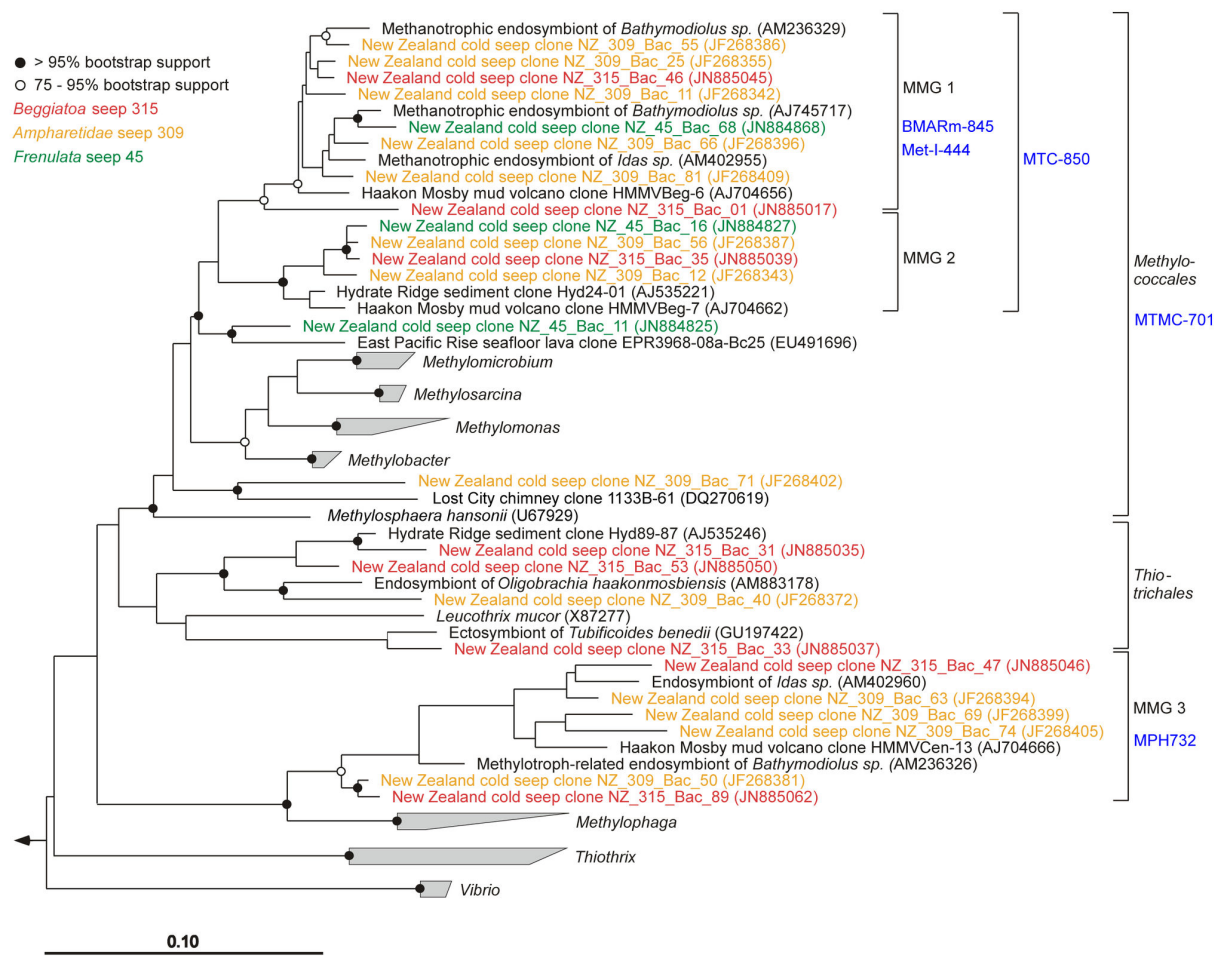
Phylogenetic affiliation of the Marine Methylotrophic Groups 1-3. Phylogeny of the Marine Methylotrophic Groups 1-3 within the *Gammaproteobacteria* based on their 16S rRNA genes. The colors indicate the origin of the sequences. Organisms belonging to the depicted groups were only found at cold seeps and not at the reference site. Probes specific for certain groups are marked in blue (see also Table S2 in the Materials and Methods S1).

Major differences between the gene libraries were observed for the *Gammaproteobacteria*. Sequences related to *Methylococcales*, *Methylophaga* and *Leucothrix* occurred in high diversity at the ampharetid sites, some were present at the frenulate site, but they were absent at the reference site. Most sequences related to methylotrophs clustered within three distinct groups that we termed Marine Methylotrophic Group 1 to Marine Methylotrophic Group 3 (MMG1-MMG3) (Figure 4). MMG1 and MMG2 are related to *Methylococcales*, while MMG3 is related to the deep-branching *Methylophaga* . Sequences of the MMG1 and MMG2 shared >95% similarity, whereas MMG3 was wider with pairwise similarities above 90%. All MMGs contained only sequences from marine samples, mostly from cold seep sediments. Additionally, MMG1 and MMG3 contained many sequences from marine methylotrophic endosymbionts of e.g. *Bathymodiolus* spp. and *Idas* spp.

### Diversity of pmoA genes

To further investigate the diversity of aerobic methanotrophic bacteria, we analyzed *pmoA* genes encoding for subunit A of the particulate methane monooxygenase. This enzyme plays a central role in aerobic methanotrophy as it activates methane by the addition of oxygen. Analysis of the top 10 cm of sediment at sites 45, 309 and 315 yielded 236 sequences and 24 OTUs. The Chao1 estimated richness was 29 OTUs. The OTUs were calculated using a cut-off of 93% amino acid sequence similarity, which corresponds to a 97% 16S rRNA similarity [46]. We found a highly diverse *pmoA* composition at the frenulate site 45 (20 OTUs). Many of the OTUs were related to endosymbionts of *Lamellibrachia* sp. and did not occur at the other seeps. The *pmoA* diversity at the SOB site 315 was also quite high (8 OTUs), but the OTUs mostly differed from the ones present at the frenulate site. Interestingly, at the ampharetid site 309 we discovered only 2 different OTUs. Both OTUs were closely related to methanotrophic endosymbionts. The *pmoA* sequences formed five very distinct monophyletic clusters (Figure S4). All clusters contained sequences exclusively from marine methane-rich ecosystems. One cluster included the cultured organism *Methylomonas methanica*. Four clusters contained sequences of either methanotrophic endosymbionts of *Bathymodiolus* spp., *Rimicaris* spp. or *Lamellibrachia* spp.

### In situ abundance and distribution of microorganisms

Abundance and distribution of microbial cells were determined for two ampharetid sites (124,309), for one SOB site (315) and one frenulate site (45). The percentages of subgroups are given relative to the total number of cells, including aggregates. Total single cell numbers (Figure 2) do not contain aggregated cells.

#### Frenulate site 45

At the site populated by frenulates, the highest cell abundance was found at 13.5 cmbsf with 2.2 × 10^9^ cells ml^-1^ sediment (Figure 2). Single cells of *Bacteria* and *Archaea* were found throughout the sediment, whereas consortia of archaeal and bacterial cells were only detected between 11 and 12 cm depth. Single cells of ANME-2 and ANME-3 were found in low numbers in the deeper sediment horizons, whereas ANME-1 was not detected at all. *Methylococcales* peaked in the top cmbsf at 4.9 × 10^7^ cells ml^-1^ sediment, but were found down to 15 cmbsf.

#### Ampharetid sites 124 and 309

**Figure 5:**
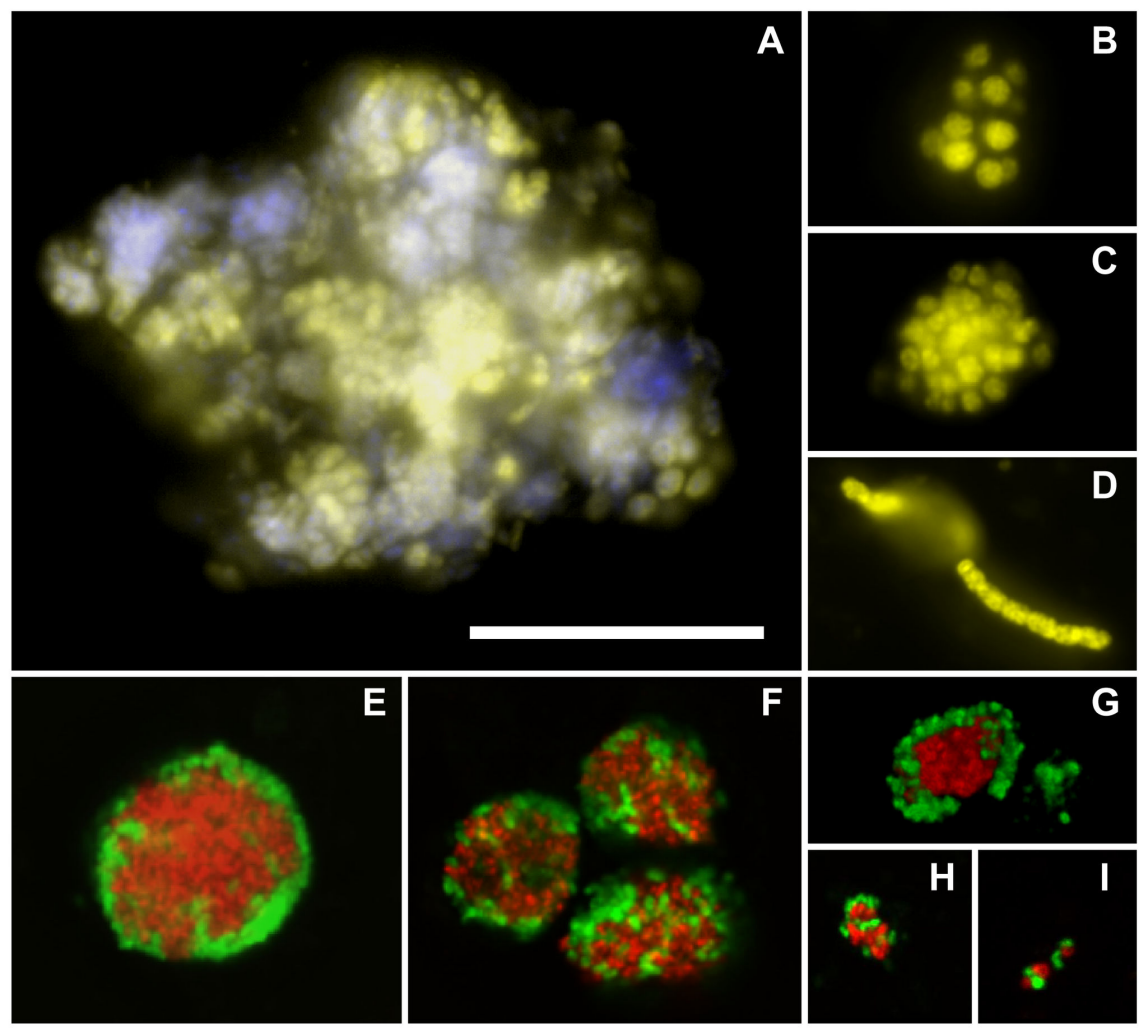
Micrographs of methylotrophic and methanotrophic organisms in sediments of Hikurangi margin seeps. **A**–**D**: Aerobic methylotrophic organisms of the order *Methylococcales* (probe MTMC-701 - yellow) in surface sediment of the ampharetid site 309. The dual stain with probe and DAPI (blue) shows that the large aggregates contained other cells besides *Methylococcales*. **E**–**I**: Consortia of anaerobic methanotrophic archaea of the clade ANME-2a and ANME2c (probe ANME2a-647, ANME2c-760 - red) and sulfate-reducing *Desulfosarcina/Desulfococcus* (DSS) (probe DSS658 - green) in the deeper sediment layers. **E**,**F**: Shell-type and mixed-type ANME-2a/DSS consortia at the SOB site 315. **G**,**H**: Consortia of ANME-2a and the DSS subgroup SEEP-SRB-1a (probe SEEP1a-473 – green) at the ampharetid site 124. **I**: ANME-2c/SEEP-SRB-1a consortium at ampharetid site 124. The scale bar represents 20 µm.

The highest total cell numbers at the ampharetid sites were found in the oxic top centimeter. Most cells were identified as *Bacteria* (80-90%, probe EUB338-I-III; LEN338), of which *Methylococcales* (probe MTMC-701 and competitors) dominated the surface sediment at both sites (12-15%). However, as many of the observed *Methylococcales* aggregated in clusters of irregular shape, their total abundance was certainly underestimated. *Methylococcales* aggregates were between 5 and 40 µm in diameter (Figure 5A–D). The large aggregates were comprised of large oval cells and appeared to contain also other cell types (Figure 5A). Small aggregates consisted of small densely packed or large loosely packed cocci (Figure 5B,C; see also Movie S1). Many aggregates consisted of cells belonging to clade MMG2 (probe MTC-850), which seemed to be responsible for most of the biomass of aerobic methylotrophs at the ampharetid habitats. The *Methylococcales* probe (MTMC-701) also visualized a few up to 30 µm long filaments with square-shaped cells (Figure 5D). Single *Methylococcales* cells occurred as rods (<1µm) and oval or round cocci (up to 2µm) showing a high morphological diversity. MMG1 (probe MetI-444) comprised cells that were stained by a probe specific for *Bathymodiolus* spp. endosymbionts (BMARm-845, Figure S5). MMG1 and MMG3 (probe MPH-732) occurred mainly in the top centimeter as single cells and at low abundances (~1-4%). Although the abundance of *Methylococcales* decreased with increasing sediment depth, MMG2 were detected as deep as 7 cmbsf. Organisms likely involved in sulfur cycling included *Epsilonproteobacteria* (~3%, probe EPSI682) and potential sulfur-oxidizing *Gammaproteobacteria* (~3%, probe Gam660) that occurred in the oxic surface layer. Single cells of sulfate-reducing *Desulfosarcina* */Desulfococcus* (probe DSS658) occurred throughout the sediment (1-14%) and peaked in the anoxic layer.

*Archaea* were almost absent in the upper oxic centimeters, but their cell numbers increased with depth. At site 124, AOM consortia of ANME-2a (probe ANME2a-647) and SEEP-SRB1a (probe SEEP1a-473) were largest and most abundant between 7 and 13 cmbsf, peaking with 4.2 × 10^7^ consortia ml^-1^ sediment (see also Movie S2). Consortia of ANME-2c (probe ANME2c-760) and SEEP-SRB1a (Figure 5I) were also detected, but in lower numbers. The diameter of the aggregates ranged from 2 µm, harboring as few as 6 cells, to 12 µm (about 10^4^ cells) with an average of 2.9 ± 0.3 µm (about 140 cells). Hence, the total number of AOM-mediating archaeal and bacterial cells at this site may have been as high as 5.9 × 10^9^ ml^-1^. We found many small AOM aggregates, lesser medium sized ones and only few large aggregates (Figure S6). At site 309, ANME-2c archaea were abundant only in around 15 cmbsf, accounting for up to 25% of all cells. They mostly occurred in small, loosely associated aggregates with DSS (Figure 5E–H). Although ANME-3 archaea were present in the 16S rRNA gene libraries they were not detected in situ (probe ANME3-1249 and helper probes). In accordance with the gene libraries, ANME-1 archaea (probe ANME1-350) were not detected. Methanogenic archaea were present in low numbers (up to 1%, probe MS1414) only in the upper horizon.

#### Sulfide oxidizing mats site 315

The sulfidic top centimeter of sediment at the SOB site also harbored the largest number of cells with *Bacteria* accounting for around 90%. In contrast to the ampharetid sites *Methylococcales* accounted only for around 1% of the community (9 × 10^7^ cells ml^-1^). Similar to site 309 we again found about 1% MMG1 and 1% MMG3, while MMG2 was almost absent. Cell numbers of aerobic methylotrophs decreased within the first 3 cmbsf and were not detected in the deeper sediment. The bacterial community of the top layer included organisms involved in sulfur cycling, such as potential sulfur-oxidizing *Gammaproteobacteria* (up to 3%) and *Epsilonproteobacteria* (up to 4%). Single cells of the DSS group occurred in all depths (up to 12%).

ANME-2 archaea were omnipresent and mostly associated with DSS. Relative abundance of ANME-2 (15% of total cells) and DSS (30%) peaked between 2 and 8 cmbsf. Single cells of ANME-2 (up to 4%) and DSS (up to 15%) were also abundant. We found the highest aggregate numbers at 2 cmbsf, whereas the largest aggregates, with diameters of >16 µm, occurred at 6 cmbsf. In the uppermost layers, ANME-2a/DSS aggregates dominated, whereas in the deeper sediment, ANME-2a and ANME-2c were equally abundant. Neither ANME-1 nor ANME-3 archaea were detected. Methanogenic archaea (probe MS1414) made up 2-3% of all cells in the horizon between 2 to 9 cm, but were absent above and below.

### Variation of microbial diversity

**Figure 6:**
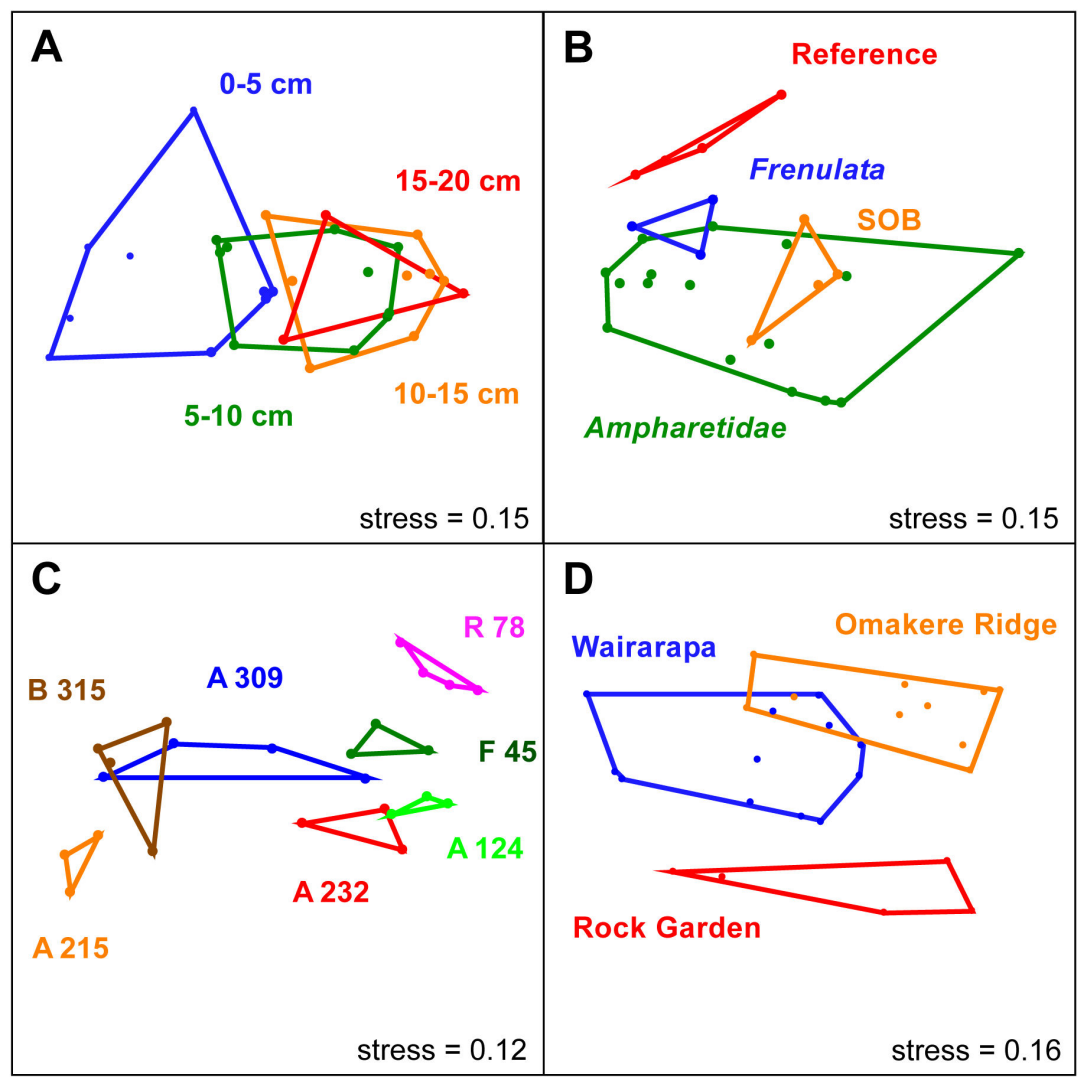
NMDS ordination plots. 3D-NMDS ordination plots, shown as 2D graphs (other axes are not shown), visualizing the ARISA dataset. The subgroups that were analyzed for each condition are depicted as colored polygons. **A**: Ordination plot of investigated depth layers. ANOSIM was used to test whether the layers are significantly different. Layer 1 (0-5 cm) was different from layer 2 (5-10 cm; R_1/2_ = 0.28, p_1/2_ = 0.013) and layer 3 (R_1/3_ = 0.6, p_1/3_ = 0.001). Layer 2 and 3 were not significantly different. We excluded the deepest layer due to its insufficient number of data points. **B**: Ordination plot of seep-associated microbial or faunal communities. ANOSIM was not performed, due to unequal group sizes. **C**: Ordination plot of sampling sites. A: *Ampharetidae*, B:SOB, F: *Frenulata*, R: Reference. Dissimilarity of the sites is supported by an R value of 0.48 (p <0.001). A 157 and A 258, as well as the bottom layers (15-20 cm) of site R 78, A 309 and B 315 were excluded from the ANOSIM to ensure equal group sizes. **D**: Ordination plot of the sampling areas Omakere Ridge (OR), Wairarapa (W) and Rock Garden (RG). The overall differences between the sampling areas are supported by an ANOSIM R value of 0.542 (p<0.001). R and p values comparing the seep areas are as follows. R_RG/OR_ = 0.7 (p<0.001); R_RG/W_ = 0.64 (p<0.001), R_W/OR_ = 0.39(p<0.001).

To get a broader perspective of the microbial communities on Hikurangi margin we assessed the variation of microbial diversity between different seep sediments by ARISA. In total 385 different OTUs (individual ITS regions of bacteria that differ in length by at least 2 bases) were found (Figure S7). Each 5 cm depth interval contained on average 149 ± 15 OTUs (mean ± S.D.). The community structure of the upper oxic/suboxic sediment (0-5 cm) was significantly different from the deeper sediment (5-15 cm) as shown by NMDS ordination and ANOSIM (Figure 6A). However, OTU partitioning according to depth showed that the majority of OTUs (e.g. 80 at ampharetid site 232) was not restricted to a certain layer, but seemed rather dispersed throughout the sediment (Figure S8A). The largest number of layer-specific OTUs always occurred in the oxic top sediment layer, and the top and bottom layers frequently shared the least OTU numbers. 51 OTUs were common to all seep sites, whereas only 7 OTUs were common to all layers at all seep sites.

Grouping the dataset according to the habitats, i.e. the dominant seep-associated fauna, showed the differences between all seep habitats and the reference site (Figure 6B). 169 OTUs were found in all three seep habitats of which 138 might be considered ubiquitous residents of Hikurangi sediments since they were also present at the reference site. Ampharetid sites shared 49 OTUs with the SOB and 69 with the frenulate site, whereas only 1 OTU was shared between the SOB and the frenulate site (Figure S8B). Thus, ampharetid habitats appear to host communities partially overlapping with those of sulfidic sediments (e.g. SOB site) and non-sulfidic sediments (reference site). Habitat type had a significant impact (p=0.02) on the microbial community as calculated by redundancy analysis (Figure S10).

Although each seep ecosystem contained a comparable number of OTUs (225 ± 30), their OTU composition differed significantly (Figure 6C). The high diversity of Hikurangi ecosystems is supported by a pairwise OTU comparison, which showed that only 55 ± 4% of OTUs are shared between any two sampling sites (Figure S9). The differences in bacterial communities were also apparent on a larger scale when we examined the sampling areas Rock Garden, Omakere Ridge and Wairarapa. The areas hosted significantly different microbial communities (Figure 6D), although more than 60% of the OTUs were shared. Interestingly, despite the larger spatial distance Omakere Ridge seeps shared many more OTUs with Wairarapa, than with Rock Garden seeps (Figure S8C). The differences in microbial diversity between the three seep areas were confirmed by RDA (p=0.003) (Figure S10).

## Discussion

### The role of ampharetid polychaetes for aerobic methanotrophy at Hikurangi margin cold seeps

Many of the cold seeps at Hikurangi margin investigated during expedition SO-191 leg 2 and 3 were densely populated by heterotrophic ampharetid polychaetes [7,8]. These occurred mainly at sites with high fluid fluxes [2,7] and with TOU rates that were among the highest reported from cold seep ecosystems worldwide, peaking at 118 mmol m^-2^ d^-1^ (mean = 84 mmol m^-2^ d^-1^) [7,10,11,12]. However, in contrast to most other seeps pore water profiles indicated that oxygen consumption was not only coupled to sulfide oxidation, but also to methane oxidation. At ampharetid habitats the bioirrigation by the polychaetes appeared to maintain an oxic/suboxic zone of about 2-4 cm, with low concentrations of sulfide. Hence, both aerobic sulfide- and methane-oxidizing microorganisms are key players in those sediments. We calculated that the aerobic community was responsible for 10-25% of the methane consumption at these seeps.

In turn, the heterotrophic polychaetes seemed to feed mainly on the methanotrophs, since the ^13^C-depleted carbon isotopic signature of their tissue clearly revealed a methane-based nutrition [7]. Infaunal density and biomass at the ampharetid habitats were among the highest that have ever been reported for cold seep sediments [47]. Thus, it appears that ampharetids and aerobic methanotrophs mutually benefit from their presence at these high fluid flux seep sites. Due to this close trophic link and the large biomass of aerobic methylotrophs, we presume that ampharetids ‘garden’ the sediment to reduce oxygen limitation in the sediment and ensure rapid growth of their food source, the aerobic methylotrophs. Formation of large cell aggregates and long filaments by the methanotrophic bacteria might therefore be explained both by a high supply of methane and oxygen, but also as a consequence of reduced grazing pressure on large cell clumps by the suspension feeding ampharetids.

### Aerobic and anaerobic methanotrophs at ampharetid habitats

We confirmed a large community of aerobic methanotrophic bacteria in the uppermost oxic-suboxic sediment layers at ampharetid habitats, whereas anaerobic methane-oxidizing archaea dominated the deeper anoxic and sulfidic layers. The aerobic methanotrophs clustered into three monophyletic clades, which we named Marine Methylotrophic Group (MMG) 1-3, because they contain sequences retrieved from marine methane-rich ecosystems or methanotrophic endosymbionts. Sequences of *Methylococcales* and MMG1 were reported from oxygen-depleted sediment [48] and *pmoA* sequences related to the ones we observed in ampharetid habitats were found in oxygen minimum zones of the Eastern Pacific Ocean [49]. Thus, methylotrophs of these clusters might either be microaerophilic or able to cope with anoxia in the deeper sediment layers. Methylotrophic bacteria affiliated with the MMG clusters have been observed as dominant populations in freshly exposed mud flows of Håkon Mosby mud volcano (HMMV) [14], and were also detected as rare members of cold seep communities by FISH [50]. MMG1 and MMG2 belong to the order *Methylococcales* while MMG3 is closely related to the deep-branching *Methylophaga* , which are non-methane utilizing methylotrophs. MMG1 are so closely related to endosymbionts of *Bathymodiolus* spp. that we visualized the cells in the sediment with a *Bathymodiolus* endosymbiont specific probe. MMG1-3 occurred also at the SOB habitat, whereas MMG3 was missing at the frenulate habitat.

The analysis of aerobic methanotrophs on a functional level, using the *pmoA* gene, revealed five monophyletic clusters that exclusively contained sequences originating from marine methane-rich ecosystems. The high diversity of *pmoA* sequences present at the SOB and frenulate habitats supported the results of the 16S rRNA gene analysis. We found organisms related to benthic and pelagic methanotrophs at both ecosystems and a high number of OTUs related to siboglinid endosymbionts at the frenulate habitat. However, the ampharetid habitat had a much lower *pmoA* diversity as was expected from the 16S rRNA analysis. All *pmoA* sequences clustered into two OTUs (at a 93% similarity cut-off) forming two distinct clades. One clade contained *pmoA* sequences related to *Bathymodiolus* spp. endosymbionts, while the other contained sequences related to *Rimicaris* sp. epibionts. The OTUs likely belonged to MMG1 or MMG2, since members of the MMG3 might lack a *pmoA* gene like their close relatives of the genus *Methylophaga* [51]. The low *pmoA* diversity might be due to a PCR bias caused by a clear dominance of these two OTUs in the sediment. MMG2 strongly dominated the methylotrophic community and contributed by far most of the biomass in the form of large aggregates. Thus, only two taxa seem to be responsible for most of the aerobic methanotrophy at ampharetid habitats despite the high diversity of methylotrophic communities found in Hikurangi ecosystems. This low diversity could be the result of a natural enrichment of aggregate-forming organisms of MMG2 caused by the polychaete activity. The existence of a specific niche at ampharetid habitats is supported by the absence of alphaproteobacterial type-II methanotrophs. We did not find them in 16S rRNA and *pmoA* gene libraries from the investigated cold seeps, although they are abundant in nearby methane-bearing sediments [52].

The anaerobic microbial communities at ampharetid habitats were confined to sulfidic sediment layers below the oxygenated zone. They were dominated by ANME-2/DSS consortia known to mediate AOM [9]. Although we found ANME-3 in the gene libraries of all seeps, in situ hybridization was only successful at frenulate site 45, which indicates that ANME-3 cells might be inactive under given conditions. ANME-1 archaea were not detected with either method. The absence of ANME-1 in bioirrigated sediments supports the hypothesis that this clade might be more sensitive to oxygen than other types [53] or it could be a result of the remoteness of the Hikurangi seep sites. Archaeal diversity and aggregate numbers as well as AOM rates were comparable to other seeps with medium to high fluid fluxes [53,54,55]. However, the AOM zone was shifted downwards compared to most other active seep sites, which is presumably due to bioirrigation by the ampharetids. An even deeper shift of the AOM zone, up to several decimeters, was observed at the frenulate habitat and other seep sediments populated by thin siboglinid tubeworms [56,57]. In contrast, at the more sulfidic habitat covered by SOB and only very few ampharetids, the diversity and abundance of aerobic methylotrophs was lower and the anaerobic methanotrophic community extended to the top sediment horizon. For comparison, at the SOB habitat anaerobic oxidation of methane and sulfate reduction rates were about 7-fold higher than at the ampharetid habitats. Biogeochemical profiles as well as diversity and biomass of ANME clades were very similar to previously described SOB sites [53,58,59]. The relationship between abundance and bioirrigation activity of ampharetids and the biogeochemical zonation of underlying sediments is an interesting future research subject.

### Comparison of Hikurangi margin seeps to previously described ecosystems

Hikurangi margin cold seeps are far away from all studied seep areas and only few deep-sea chemosynthetic ecosystems are known from the Southern Hemisphere [60,61,62]. Previous studies have found very strong and long lasting seepage activity [63] and substantial differences concerning the composition and diversity of the local seep fauna [6]. Here we investigated the seep microbial communities and compared them to those from the Northern Hemisphere. Representatives of the main functional groups, such as aerobic and anaerobic methanotrophs, sulfide oxidizers and sulfate reducers of Hikurangi margin were closely related to those known from other seeps described before. However, the biogeochemistry and occurrence of methylotrophic groups was different. Especially the diversity and abundance of aerobic methylotrophs is to our knowledge unprecedented. Another outstanding feature of these seeps is the absence of ANME-1 at all investigated sediments. To understand whether this is a result of geographic remoteness or due to metabolic constraints of this clade we need detailed analyses of other ampharetid habitats, such as the ones recently discovered at the Makran margin [56]. An overview of important biogeochemical and microbial characteristics of Hikurangi margin cold seeps, compared to sites from Hydrate Ridge, HMMV and the Black Sea is provided (Table 2 and Table S1).

**Table 2:** Biogeochemical and physical characteristics of Hikurangi margin, Hydrate Ridge, Black Sea and Håkon Mosby mud volcano seep sites.

	MOx rates (µmol cm^-3^ day^-1^)	SR rates (µmol cm^-3^ day^-1^)	Temp. (°C)	Water depth (m)	Oxygen penetration (mm)	Sulfate conc. (mM)	Sulfide conc. (mM)	Methane conc. (µM)	Methane flux (mmol m^-2^ day^-1^)
Hikurangi *Frenulata* site 45	n.a.	<0.15	4-5	1159	n.a.	29	0	2-4	n.a.
Hikurangi *Ampharetidae* site 124	0.02-0.2	0.05-0.35	4-5	1054	1-4^#^	5-29	0-15	80-530	>200^#^
Hikurangi *Ampharetidae* site 309	0.1-0.25	0.05-0.2	4-5	1057	1-4^#^	3-26	n.a.	10-600	>200^#^
Hikurangi *Beggiatoa* site 315	0.1-0.5	0.1-1.2	4-5	1057	n.a.	0-23	n.a.	15-360	n.a.
Hydrate Ridge *Beggiatoa*	<3	<2.1	2-4	777	1	<18	10-26	n.a.	30-90
Hydrate Ridge *Calyptogena*	<2.7	<3.6	2-4	787	10	18-26	0-10	n.a.	<1
Black Sea sediment P817	0.4-0.7	1.4-2.1	8-9	192	anoxic	10-13	1	80-150	n.a.
Black Sea P822 microbial mat inside chimney	300*	n.a.	8-9	191	anoxic	6	3	4000	n.a.
HMMV *Beggiatoa* site	0.1-0.6	0.1-1	-1	1250	1-2	0-30	0-4	0.3**	50-150***
HMMV tubeworm site	0-0.25	0-0.25	-1	1250	30-100	25-30	0	0.7**	50-150***

^#^ average for ampharetid sites * µmol g dry weight^- 1^ day^- 1^ ** measured above sediment *** mud volcano average n.a. not assessed

References[7,14,52,56,69,70,71,72,7]:

### Drivers of biodiversity at Hikurangi margin seep ecosystems

ARISA data revealed a comparable richness of abundant bacterial types at each site and in the four depth layers investigated. However, β-diversity was considerably different at all spatial scales from a few centimeters to hundreds of kilometers. For example, microbial communities in two depth layers of the same ecosystem frequently shared as little or less OTUs as two seep ecosystems from different geographical regions. The observed increasing dissimilarity of the community with increasing sediment depth was already described in previous studies [33,64]. This effect was also present at the reference site, and is not unique to seep ecosystems. Our results support the hypothesis that availability of electron acceptors, especially oxygen, is a main driver influencing chemosynthetic communities and biodiversity of cold seep sediments [53,56]. In contrast, no direct link was observed between methane oxidation rates, sulfate reduction rates and community composition or richness, probably because of the relatively similar range of biogeochemical activity across the different seeps investigated. However, the presence of different fauna had a significant impact on the underlying bacterial community. This supports the finding that the presence and activity of the polychaete worms shape the underlying microbial communities at ampharetid habitats. In addition, the seep area had a significant influence on the microbial communities. This might be due to a number of factors, such as differences in the geochemistry, water currents or distance to the coast. In contrast to earlier findings from the San Pedro Basin and the Laptev Sea [65,66], water depth did not shape the communities at Hikurangi. This study shows that at least for the key environmental functions like methano- and thiotrophy at cold seeps, similar types of biogeochemical settings select for similar community composition, despite the large geographical distance to known environments covered here.

## Conclusion

Hikurangi margin harbors cold seep ecosystems with unique features concerning seep-associated fauna and methanotrophic communities. Bioirrigation by the ampharetid polychaetes maintains a habitat suitable for aerobic methylotrophic bacteria in the top sediment, contributing to about 25% of total methane consumption, while anaerobic methanotrophic archaea dominate the deeper anoxic layers. Dense polychaete communities such as the ones formed by ampharetids investigated here are present also at other seepage sites [56,66,67]. Accordingly, the role of aerobic oxidation of methane in seafloor methane budgets may be underestimated.

## Supporting Information

Figure S1 **Biogeochemistry of additional Hikurangi ecosystems.**
(PDF)

Figure S2 **Phylogeny of archaeal 16S rRNA.**
(PDF)

Figure S3 **Phylogeny of deltaproteobacterial 16S rRNA.**
(PDF)

Figure S4 **Phylogeny of *pmoA* protein.**
(PDF)

Figure S5 **Micrographs of organisms related to *Bathymodiolus* spp. Endosymbionts.**
(PDF)

Figure S6 **Abundance and size distribution of AOM aggregates.**
(PDF)

Figure S7 **Number of operational taxonomic units at each site.**
(PDF)

Figure S8 **Partitioning of operational taxonomic units.**
(PDF)

Figure S9 **Shared operational taxonomic units between sites.**
(PDF)

Figure S10 **Redundancy analysis of ARISA data and environmental parameters.**
(PDF)

Movie S1 **3D animated methylococci aggregate.** The methylococci aggregate originates from the oxic layer at ampharetid site 124. The cells are hybridized with the *Methylococcales* specific probe (MTMC-701, green) and nucleic acid is stained with DAPI (blue). The aggregate consists of large oval cells that are loosely packed. For details concerning the animation see Materials and Methods S1.
(MPG)

Movie S2 **3D animated AOM aggregate.** The AOM aggregate was found in the anoxic layer of ampharetid site 124. The diameter was 12 µm, total cell volume of the aggregate (excluding intercellular space), based on the DAPI signal, was 76 µm^3^, the volume of the archaeal core (ANME-2a-647 - red) was 20 µm^3^ and the bacterial shell (SEEP1a-473 - green) was 25 µm^3^. For details concerning animation and volume calculation see Supporting Material and Methods.
(AVI)

Table S1 **Total cell counts and relative abundance of methanotrophs at Hikurangi margin, Hydrate Ridge, Black Sea and Håkon Mosby mud volcano seep sites.**
(PDF)

Materials and Methods S1 (PDF)
