# Supplementary material for: Microbial Communities of Deep-Sea Methane Seeps at Hikurangi Continental Margin (New Zealand)
Source: PLoS One. 2013 Sep 30;8(9):e72627. doi: 10.1371/journal.pone.0072627 (PMC3787109; doi:10.1371/journal.pone.0072627)
Supplement: Figure S1 — Biogeochemistry of additional Hikurangi ecosystems. (PDF) [file pone.0072627.s001.pdf]

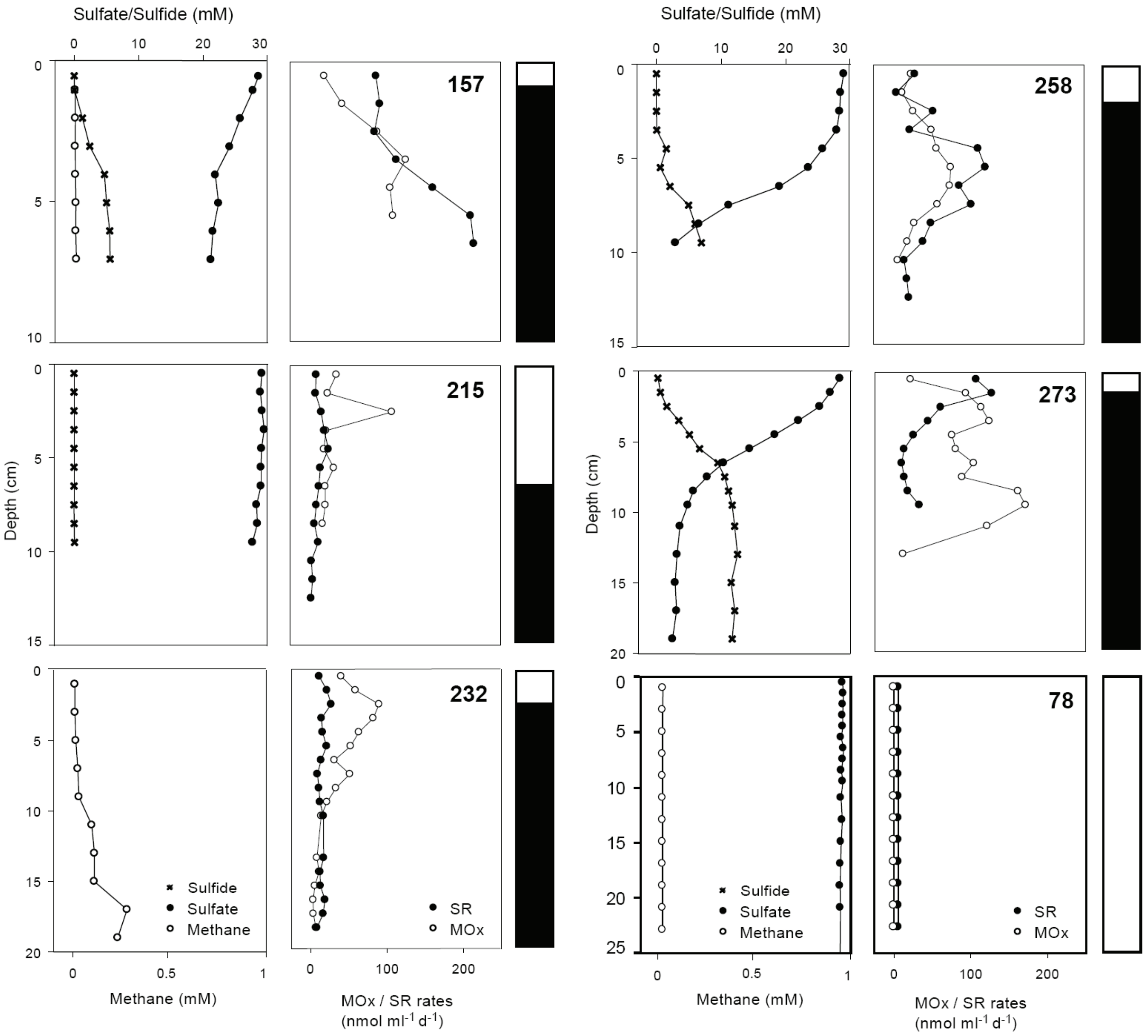

Figure S1: Biogeochemistry of additional Hikurangi ecosystems

Methane and sulfate concentrations, rates of methane oxidation (MOx) and sulfate reduction (SR) of five additional ampharetid habitats (157, 215, 232, 258 and 273) and a reference site (78) situated on Hikurangi margin. The bars on the side show the redox state of the sediment: White is the oxic/suboxic and black the anoxic/sulfidic zone. All sites show very low sulfide concentrations in the upper sediment horizons. Methane oxidation at these sites took place in the oxic and anoxic layers.
