# Supplementary material for: Microbial Communities of Deep-Sea Methane Seeps at Hikurangi Continental Margin (New Zealand)
Source: PLoS One. 2013 Sep 30;8(9):e72627. doi: 10.1371/journal.pone.0072627 (PMC3787109; doi:10.1371/journal.pone.0072627)
Supplement: Figure S2 — Phylogeny of archaeal 16S rRNA. (PDF) [file pone.0072627.s002.pdf]

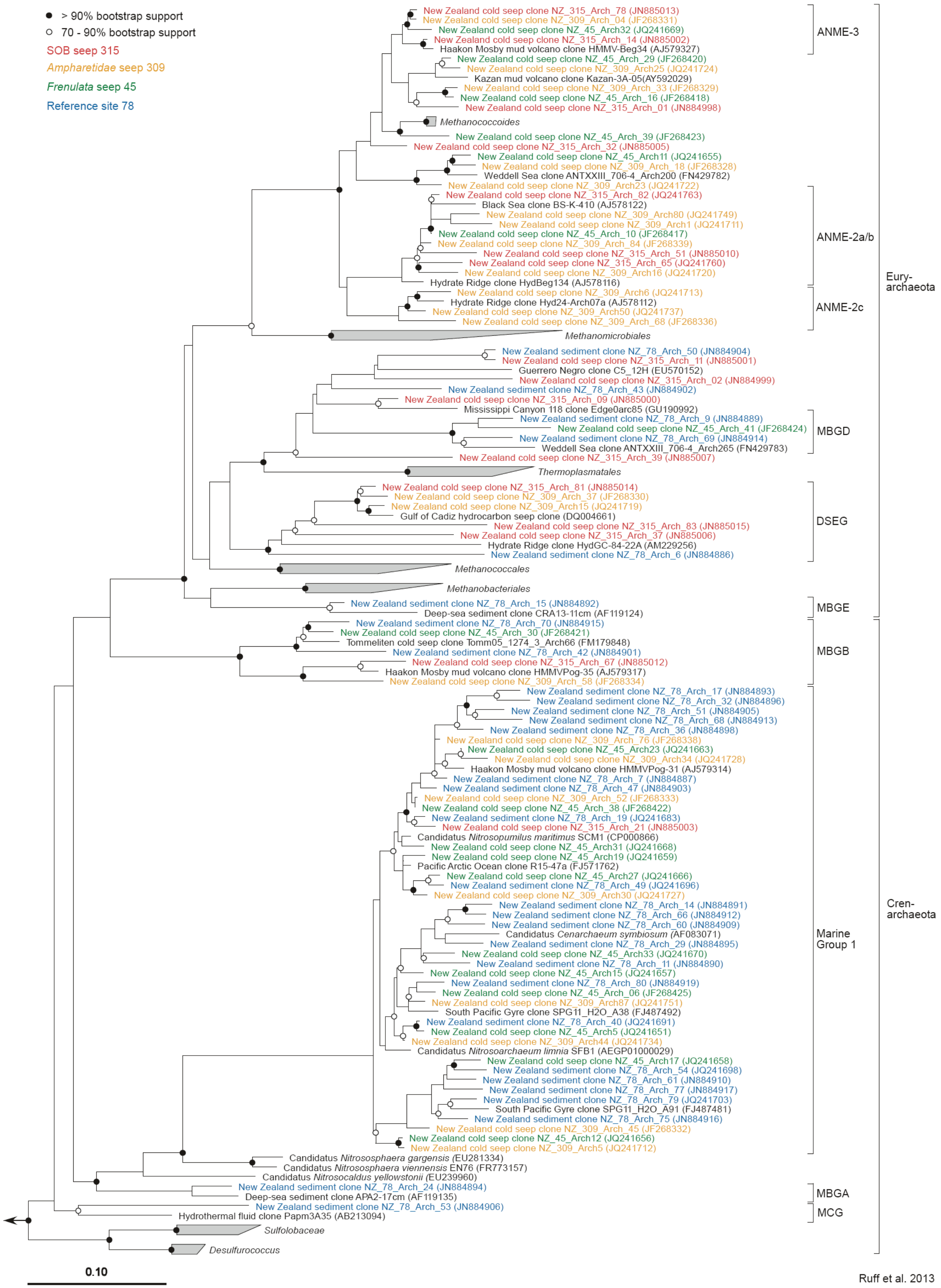

Ruff et al. 2013

Figure S2: Phylogeny of archaeal 16S rRNA

16S rRNA based phylogenetic tree showing archaeal sequences detected at the Hikurangi margin together with selected reference sequences of the domain *Archaea*. The tree is based on the RAxML algorithm as implemented in ARB. Full-length sequences were obtained from the ampharetid site 309 (yellow) and the frenulate site 45 (green). Partial sequences were retrieved from the SOB site 315 (red) and the reference site 78 (blue). Bar = 10% estimated sequence divergence.
