# Supplementary material for: Microbial Communities of Deep-Sea Methane Seeps at Hikurangi Continental Margin (New Zealand)
Source: PLoS One. 2013 Sep 30;8(9):e72627. doi: 10.1371/journal.pone.0072627 (PMC3787109; doi:10.1371/journal.pone.0072627)
Supplement: Figure S4 — Phylogeny of pmoA protein. (PDF) [file pone.0072627.s004.pdf]

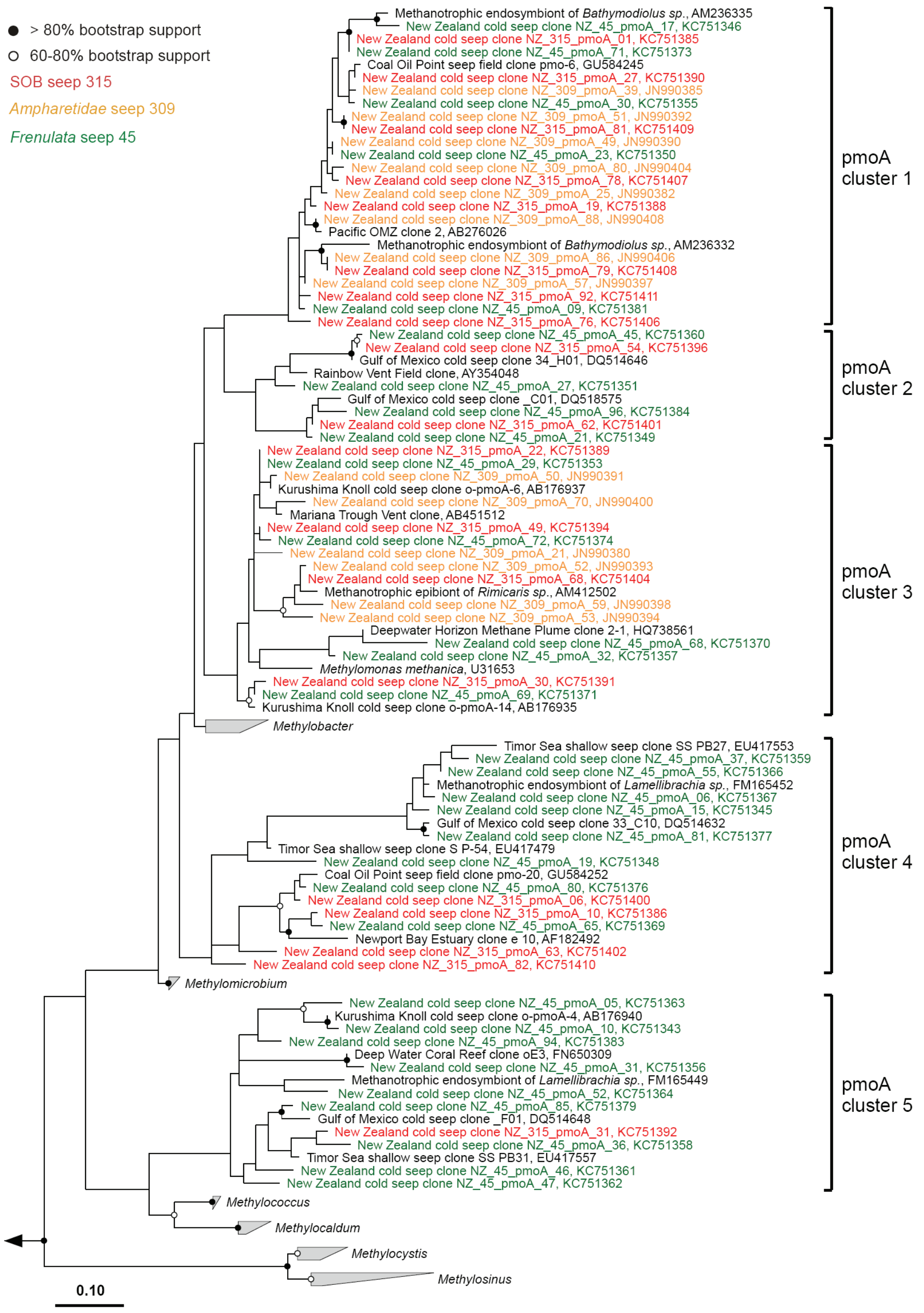

Figure S4: Phylogeny of *pmoA* protein

Phylogeny of the *pmoA* protein at the frenulate site 45, the ampharetid site 309 and the SOB site 315. The tree was calculated using 98 non-redundant, curated amino acid sequences,from the three habitats and 90 reference sequences. We used a maximum likelihood algorithm employing 100 bootstraps and a positional variability filter, excluding the highly variable regions. Bar = 10% estimated sequence divergence.
