# Supplementary material for: Microbial Communities of Deep-Sea Methane Seeps at Hikurangi Continental Margin (New Zealand)
Source: PLoS One. 2013 Sep 30;8(9):e72627. doi: 10.1371/journal.pone.0072627 (PMC3787109; doi:10.1371/journal.pone.0072627)
Supplement: Figure S5 — Micrographs of organisms related to Bathymodiolus spp. Endosymbionts. (PDF) [file pone.0072627.s005.pdf]

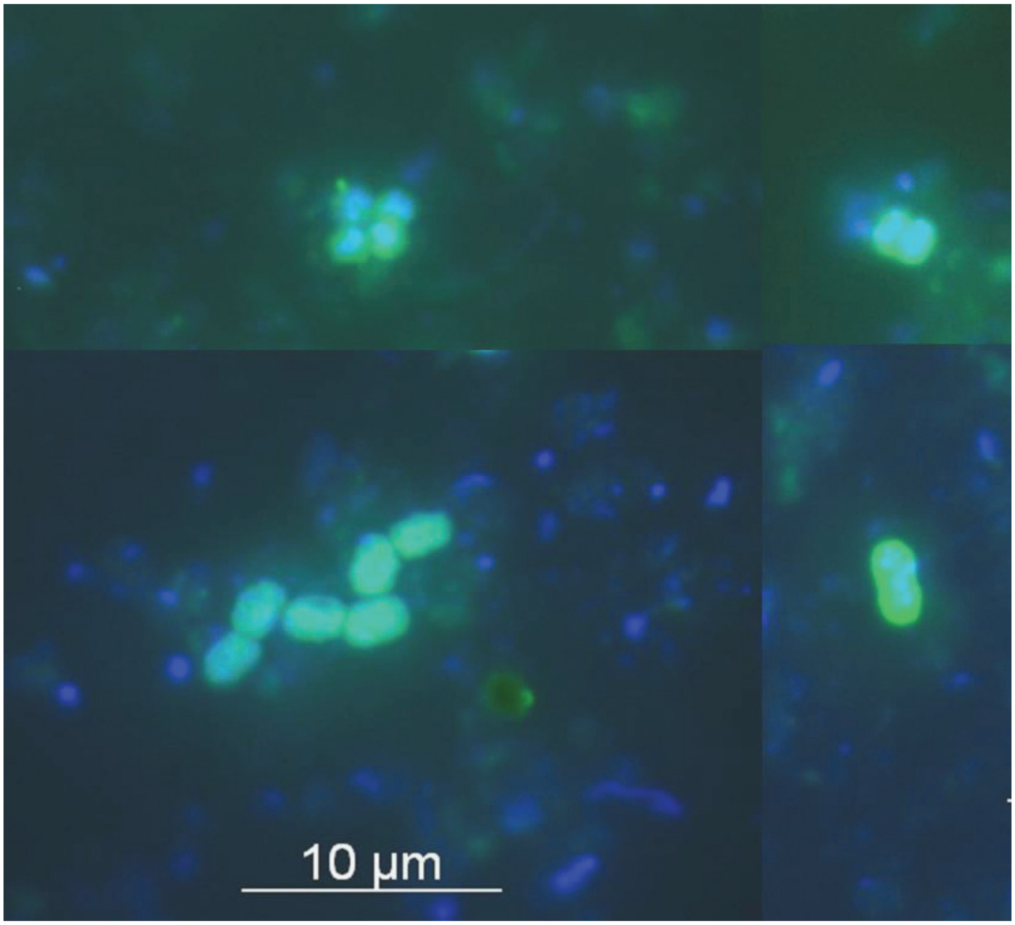

Figure S5: Micrographs of organisms related to *Bathymodiolus* spp. endosymbionts

Micrographs of MMG1 organisms in surface sediment of the ampharetid habitat (site 309). The cells were stained by the nucleic acid stain DAPI (blue) and a probe that detects the methanotrophic endosymbionts of *Bathymodiolus* spp. and other MMG1 bacteria (probe BMARm-345 - green).
