# Supplementary material for: Microbial Communities of Deep-Sea Methane Seeps at Hikurangi Continental Margin (New Zealand)
Source: PLoS One. 2013 Sep 30;8(9):e72627. doi: 10.1371/journal.pone.0072627 (PMC3787109; doi:10.1371/journal.pone.0072627)
Supplement: Figure S6 — Abundance and size distribution of AOM aggregates. (PDF) [file pone.0072627.s006.pdf]

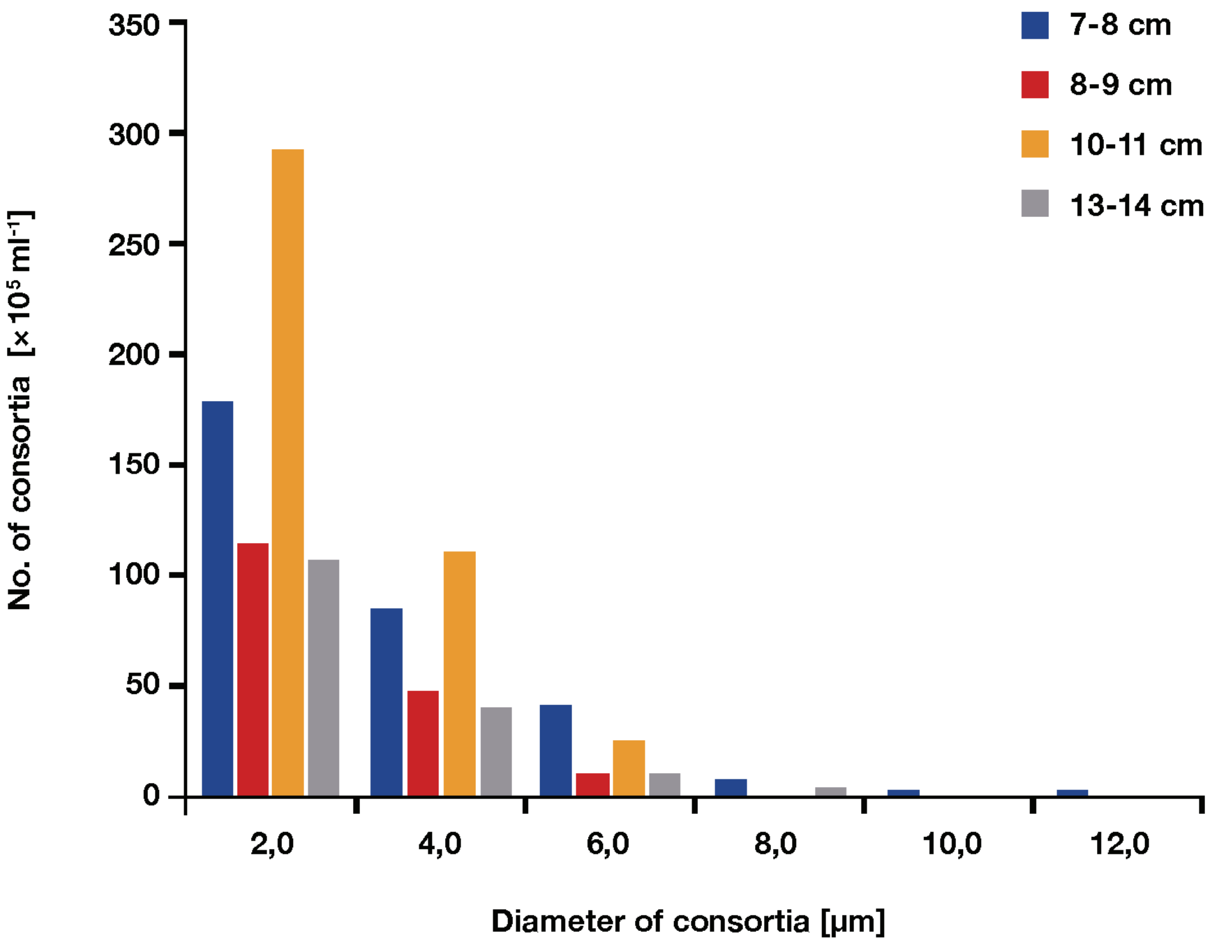

Figure S6: Abundance and size distribution of AOM aggregates

Abundance and size distribution of ANME-2a/SEEP-SRB-1a aggregates at ampharetid site 124. Bars of the same color represent consortia of different sizes in the same depth layer. In contrast to other seep sites the diameter of the aggregates is not normally distributed, instead the population is dominated by small aggregates.
