# Supplementary material for: Microbial Communities of Deep-Sea Methane Seeps at Hikurangi Continental Margin (New Zealand)
Source: PLoS One. 2013 Sep 30;8(9):e72627. doi: 10.1371/journal.pone.0072627 (PMC3787109; doi:10.1371/journal.pone.0072627)
Supplement: Figure S7 — Number of operational taxonomic units at each site. (PDF) [file pone.0072627.s007.pdf]

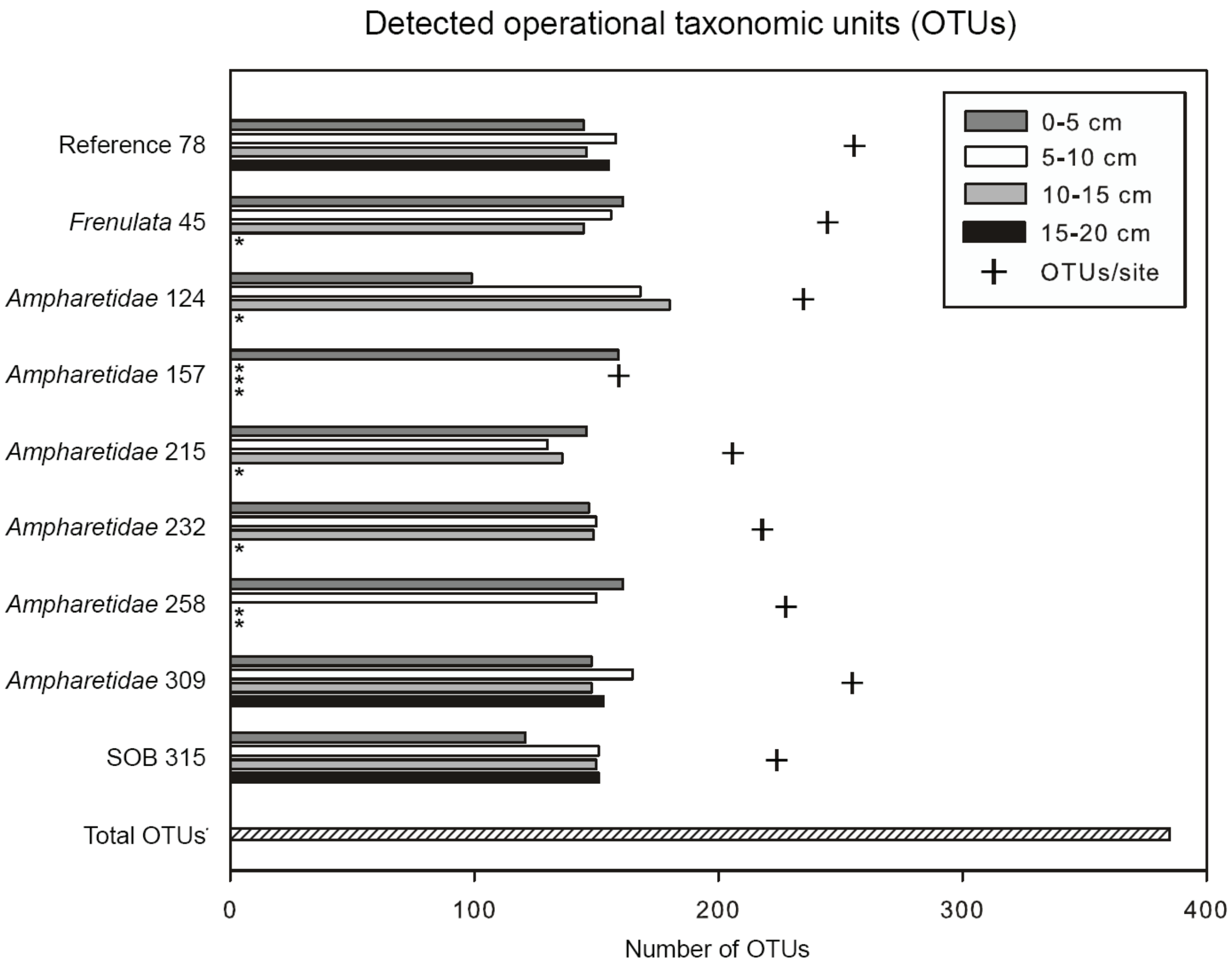

Figure S7: Number of operational taxonomic units at each site

Total number of operational taxonomic units (OTUs) present in the investigated depth layers (horizontal bar) and in total for each sampling site (crosses) as detected by the DNA fingerprinting technique ARISA. * denotes depth intervals that are missing due to shorter sediment cores. The striped bar (bottom of graph) represents the total number of unique OTUs detected on Hikurangi margin during this study.
