# Supplementary material for: Microbial Communities of Deep-Sea Methane Seeps at Hikurangi Continental Margin (New Zealand)
Source: PLoS One. 2013 Sep 30;8(9):e72627. doi: 10.1371/journal.pone.0072627 (PMC3787109; doi:10.1371/journal.pone.0072627)

Figure S8: Partitioning of operational taxonomic units

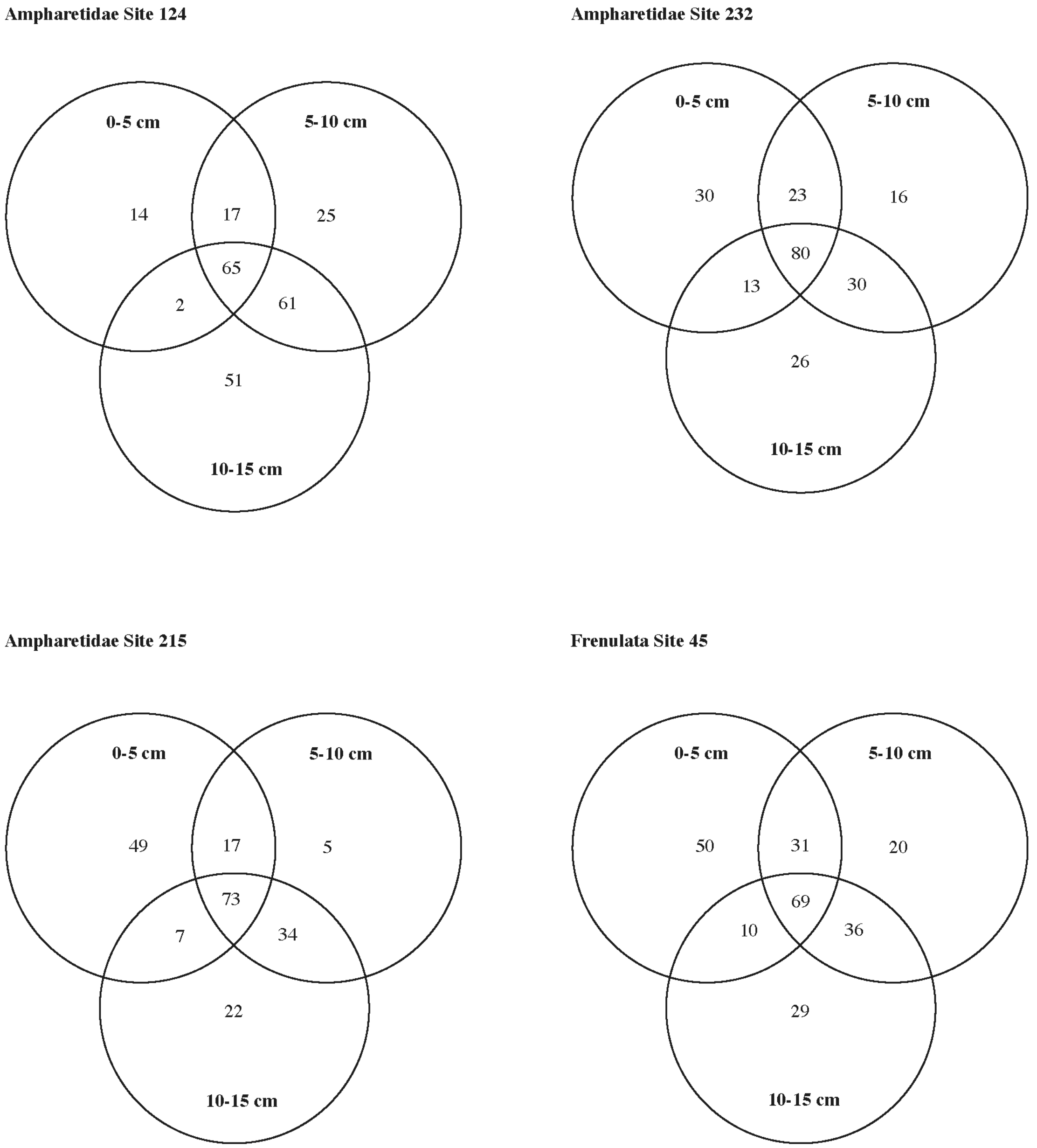

Figure S8A: OTU partitioning based on depth layers (continuing on next page)

Sites 309, 315 and 78 include four depth layers, sites 124, 215, 232 and 45 only three sediment layers. Small circles to the right of each larger graph depict two further combinations that could not be visualized in the main graph. Numbers in circles and their intersections indicate OTUs that are shared between the respective layers. The sum of all numbers equals the total number of OTUs detected at the respective site.

Ampharetidae Site 309

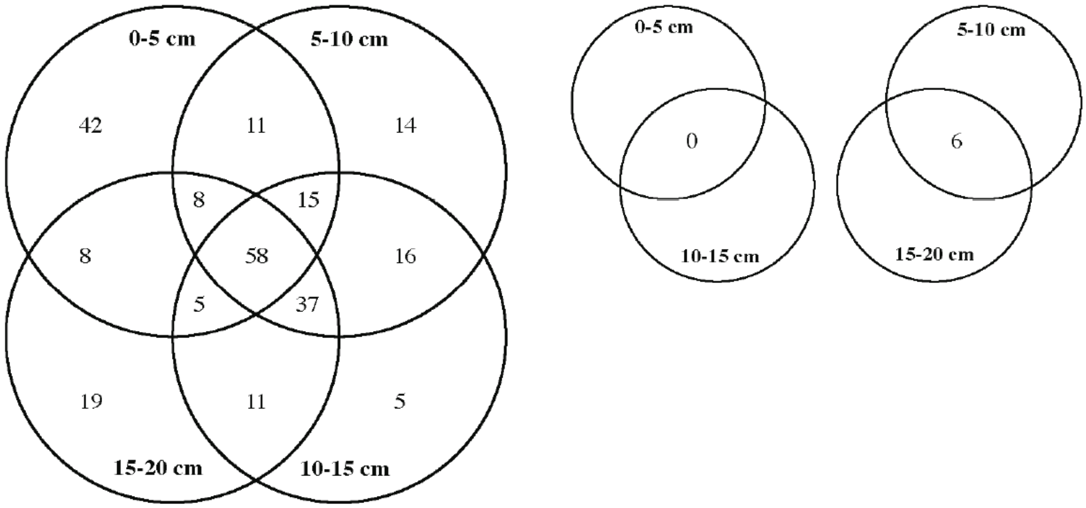

SOB Site 315

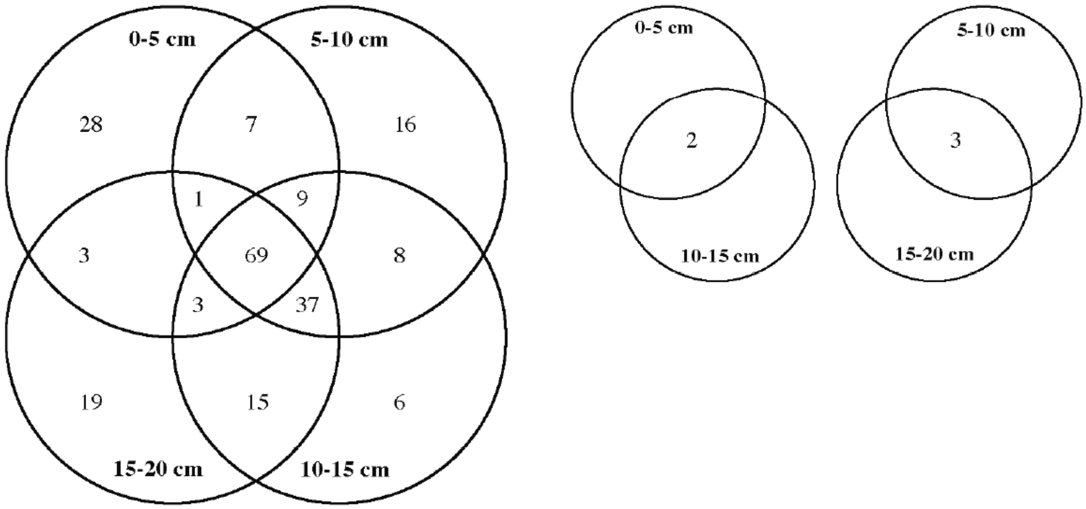

Reference Site 78

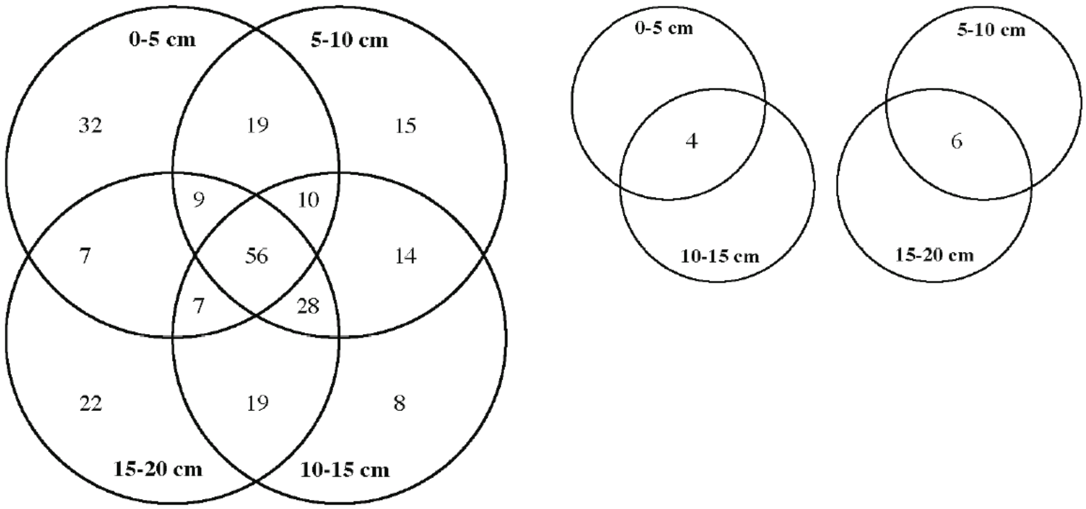

Figure S8B: OTU partitioning based on habitats

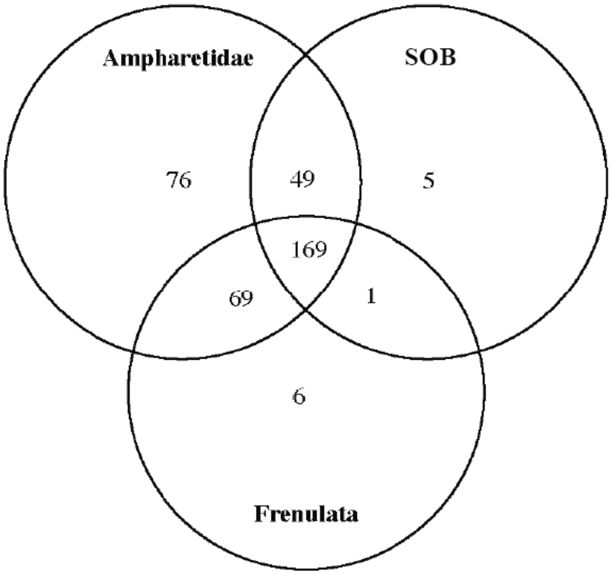

Figure S8C: OTU partitioning based on sampling areas

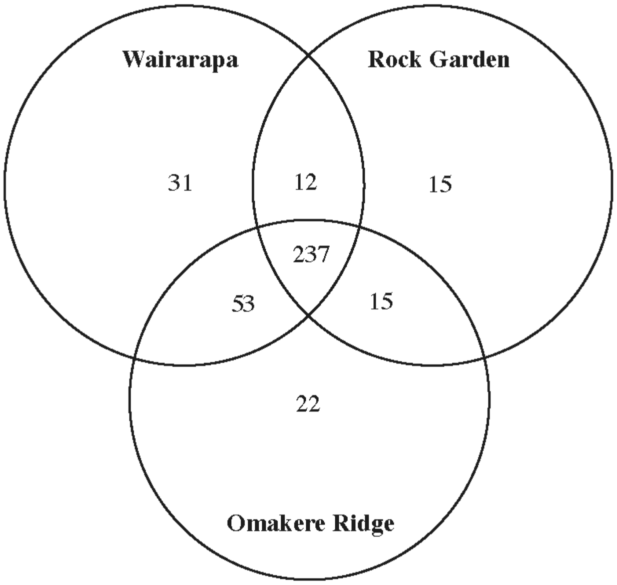

Supplement: Figure S8 — Partitioning of operational taxonomic units. (PDF) [file pone.0072627.s008.pdf]
