# Supplementary material for: Microbial Communities of Deep-Sea Methane Seeps at Hikurangi Continental Margin (New Zealand)
Source: PLoS One. 2013 Sep 30;8(9):e72627. doi: 10.1371/journal.pone.0072627 (PMC3787109; doi:10.1371/journal.pone.0072627)
Supplement: Figure S9 — Shared operational taxonomic units between sites. (PDF) [file pone.0072627.s009.pdf]

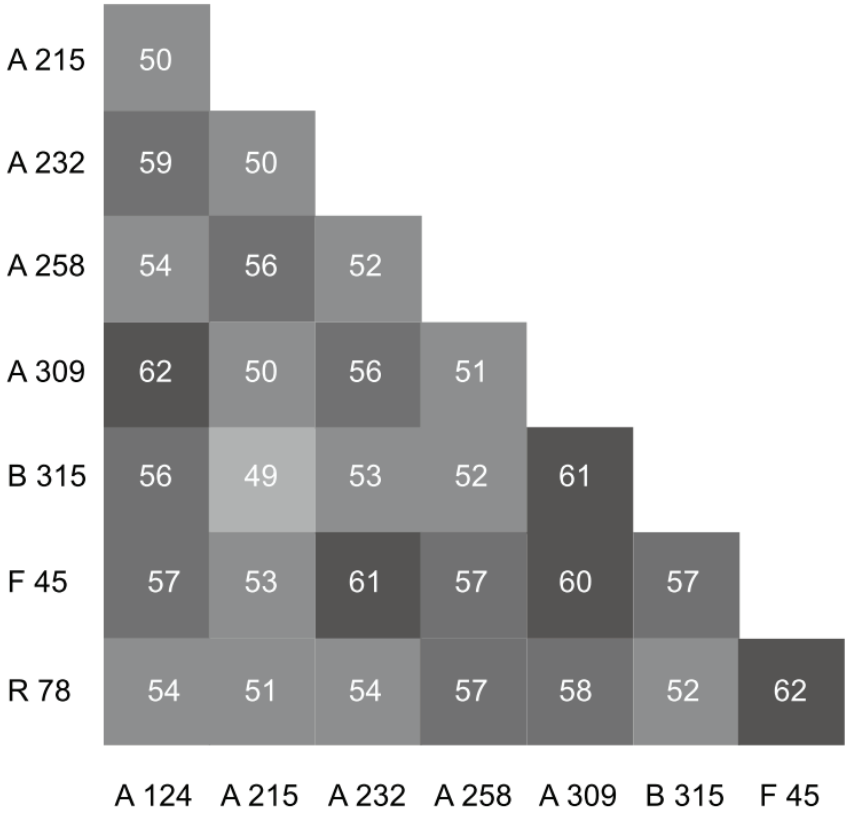

Figure S9: Shared operational taxonomic units between sites

Percentage of operational taxonomic units (OTUs) that are shared between the investigated sites. The letter in front of the station number indicates the habitat type (A = *Ampharetidae*, B = SOB, F = *Frenulata*, R = Reference).
