# Supplementary material for: Microbial Communities of Deep-Sea Methane Seeps at Hikurangi Continental Margin (New Zealand)
Source: PLoS One. 2013 Sep 30;8(9):e72627. doi: 10.1371/journal.pone.0072627 (PMC3787109; doi:10.1371/journal.pone.0072627)
Supplement: Figure S10 — Redundancy analysis of ARISA data and environmental parameters. (PDF) [file pone.0072627.s010.pdf]

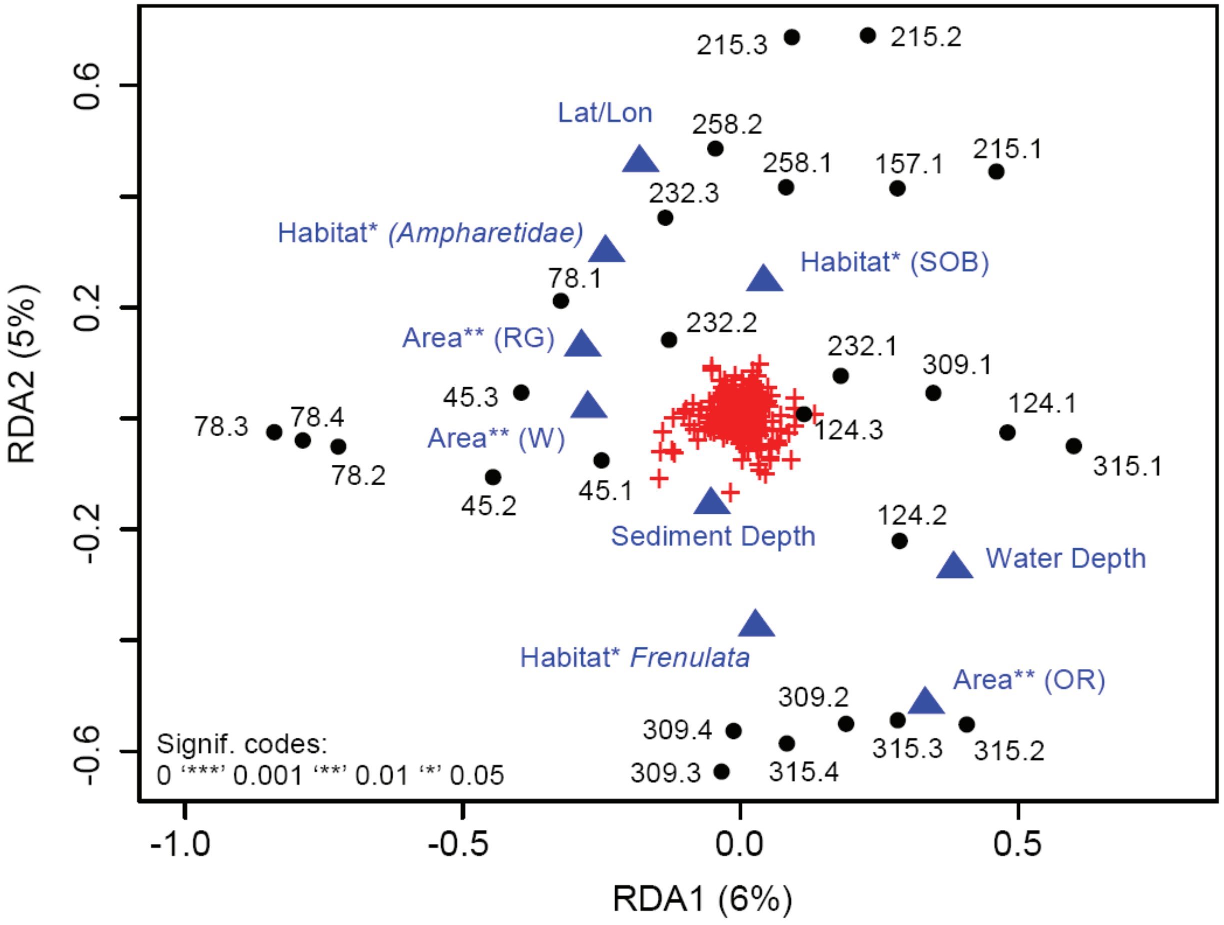


**Figure S10: Redundancy analysis of ARISA data and environmental parameters**

Redundancy analysis based on the Hellinger-transformed ARISA dataset and environmental parameters. The plot shows the full model considering all parameters, which explained 23% of the total variation (p=0.001). Black circles represent the microbial community of a given sampling site and depth layer (e.g. 124.1 = station 124, 0-5 cm; 124.2 = station 124, 5-10cm). Species are shown as red crosses, environmental parameters as blue triangles (OR: Omakere Ridge, RG: Rock Garden, W: Wairarapa). Note: In RDA plots environmental parameters and species are generally depicted as centered arrows originating in point 0/0 (which represents the average and not zero). To simplify the plot we chose to show just the tips of the arrows as crosses and triangles. Significance levels were calculated for each parameter using partial RDA and ANOVA. Only habitat type and seep area were significant on their own.
