# Supplementary material for: Microbial Communities of Deep-Sea Methane Seeps at Hikurangi Continental Margin (New Zealand)
Source: PLoS One. 2013 Sep 30;8(9):e72627. doi: 10.1371/journal.pone.0072627 (PMC3787109; doi:10.1371/journal.pone.0072627)
Supplement: Table S1 — Total cell counts and relative abundance of methanotrophs at Hikurangi margin, Hydrate Ridge, Black Sea and Håkon Mosby mud volcano seep sites. (PDF) [file pone.0072627.s013.pdf]

Table S1: Total cell counts and relative abundance of methanotrophs at Hikurangi margin, Hydrate Ridge, Black Sea and Håkon Mosby mud volcano seep sites

	**Total Cells (10^{10} cm^{3})**	***Methylococcales***	**ANME-1**	**ANME-2a/DSS Consortia**	**ANME-2c/DSS Consortia**	**ANME-3/DBB Consortia**
**Hikurangi *Ampharetidae* (124)**	0.15	+++	-	+++	+	n.a.#
**Hikurangi *Ampharetidae* (309)**	0.2	+++	-	++	++	n.a.#
**Hikurangi *Frenulata* (45)**	0.13	+	-	+	-	+
**Hikurangi SOB (315)**	1	+	-	+++	+	n.a.#
**Hydrate Ridge *Beggiatoa* site**	4.2	n.a.	+	+++	+	-
**Hydrate Ridge *Calyptogena* site**	4.6	n.a.	+	+	+++	-
**Black Sea sediment P817**	2.7	n.a.	+	+ ##	+ ##	-
**Black Sea P822 microbial mat, top part of chimney**	30	n.a.	+	+++	+	-
**Black Sea P822 microbial mat, inside of chimney**	1.4	n.a.	+++	+	+	-
**HMMV *Beggiatoa* site**	1.9	+	-	- ##	- ##	+++
**HMMV tubeworm site**	0.33	-	+	++ ##	++ ##	++

+present in low proportions ++high proportions +++dominant type n.a.: not assessed #present as 16S rRNA gene ## ANME-2/DSS

References:

Knittel et al. (2005) Diversity and Distribution of Methanotrophic Archaea at Cold Seeps. Appl Environ Microbiol 71: 467-479.
Niemann et al. (2006) Novel microbial communities of the Haakon Mosby mud volcano and their role as a methane sink. Nature 443: 854-858.
Lösekann et al. (2007) Diversity and Abundance of Aerobic and Anaerobic Methane Oxidizers at the Haakon Mosby Mud Volcano, Barents Sea. Appl Environ Microbiol 73: 3348-3362.
Schreiber et al. (2010) Identification of the dominant sulfate-reducing bacterial partner of anaerobic methanotrophs of the ANME-2 clade. Environ Microbiol 12: 2327-2340.28.
Arnds (2009) Molecular characterization of microbial populations in methane-rich marine habitats. Bremen: Universität Bremen. 158 p.
Knittel et al. (2003) Activity, distribution, and diversity of sulfate reducers and other bacteria in sediments above gas hydrate (Cascadia Margin, Oregon). Geomicrobiol J 20: 269-294.
