# Supplementary material for: Microbial Communities of Deep-Sea Methane Seeps at Hikurangi Continental Margin (New Zealand)
Source: PLoS One. 2013 Sep 30;8(9):e72627. doi: 10.1371/journal.pone.0072627 (PMC3787109; doi:10.1371/journal.pone.0072627)
Supplement: Materials and Methods S1 — (PDF) [file pone.0072627.s014.pdf]

Supporting Material and Methods

ARISA

ARISA was carried out according to Böer and colleagues [1] with the following modifications. Polymerase chain reactions (PCRs) (50 µl total volume) were conducted in triplicates containing 1× PCR buffer, 0.25 mM dNTP mix, 0.09 mg ml^{-1} bovine serum albumin, 400 nM each of universal primer ITSF (Table S1), labeled with phosphoramidite dye FAM and bacterial ITSReub (Table S1), 0.05 units MasterTaq polymerase and 20-25 ng environmental DNA. PCR was carried out in an Eppendorf MasterCycler with an initial denaturation for 3 min at 94°C, followed by 30 cycles of 94°C for 45 sec, 55°C for 45 sec, 72°C for 90 sec and a final extension for 5 min at 72°C. PCR products were purified utilizing Sephadex G-50 Superfine, which is based on the principle of gel filtration by cross-linked dextran molecules. Sephadex was filled in the wells of a Multiscreen HV plate and soaked in 300 µl of HPLC-grade water per well for 3 h at RT, excess water was removed using an Eppendorf Centrifuge 5810R (910 × *g* for 5 min at RT), the wells were washed with 150 µl HPLC water and again centrifuged (as above). PCR products were loaded in the center of each well, centrifuged (as above) and collected in a sterile 96-well plate. A standardized amount of amplified DNA (100 ng) was mixed with a separation cocktail containing 0.5 µl (when <3 µl PCR products were used) or 1 µl (when ≥3 µl PCR products were used) of internal size standard Map Marker 1000 ROX (50-1000 bp), 0.5 µl tracking dye and 14 µl of deionized Hi-Di formamide. Discrimination of the PCR-amplified fragments via capillary electrophoresis was carried out on an ABI PRISM 3130xl Genetic Analyzer (Applied Biosystems) and the ARISA profiles were analyzed using the GeneMapper Software v3.7.

OTU partitioning and pairwise comparison

OTU partitioning was used to determine the number of OTUs that are specific for a certain condition and was performed on the ARISA dataset using Microsoft Excel and a custom R script [2]. Pairwise OTU comparison was based on a custom R script.

Volume calculation and 3D animation of cell aggregates

The three dimensional imaging was done using confocal laser scanning microscopy (C-LSM) and aggregate volume was calculated with PHLIP [3]. For the 3D animation of the AOM consortium (Movie S2) we used *daime* [4], the animation of the Methylococci aggregate (Movie S1) was calculated with IMARIS (v 7.1.1, Bitplane AG, Zürich, Switzerland). Both animations are based on C-LSM picture stacks that were processed and filtered (median filter, 2 pixels radius) with Image J v1.43 (http://rsb.info.nih.gov/ij/index.html) or deconvoluted with AutoQuant (v x2.2, Media Cybernetics, USA).

Table S2: Oligonucleotide probes and primers used in this study

Oligonucleotides	Specificity	Target Gene	Target Position	FA conc. (% v/v)	Annealing (°C)	Nucleotide Sequence 5'-3'	Reference
A189F	pmoA	pmoA	189-206	-	55	GGN GAC TGG GAC TTC TGG	[5]
Arch20F	most *Archaea*	16S rRNA	20-38	-	58	TTC CGG TTG ATC CYG CCR G	[6]
GM3	*Bacteria*	16S rRNA	8-23	-	44	AGA GTT TGA TCM TGG C	[7]
GM4	*Bacteria*	16S rRNA	1492-1507	-	44	TAC CTT GTT ACG ACT T	[7]
ITSF FAM	Universal	16S rRNA	1423-1443	-	55	GTC GTA ACA AGG TAG CCG TA	Modified after [8]
ITSREub	*Bacteria*	23S rRNA	23-38	-	55	GCC AAG GCA TCC ACC	[8]
MB661R	pmoA	pmoA	661-679	-	55	CCG GMG CAA CGT CYT TAC C	[9]
Uni1392R	Universal	16S rRNA	1392-1406	-	58	ACG GGC GGT GTG TRC	[10]
ANME-1-350	ANME-1	16S rRNA	350-367	40	-	AGT TTT CGC GCC TGA TGC	[11]
ANME-2a-647	ANME-2a	16S rRNA	647-664	35	-	TCT TCC GGT CCC AAG CCT	[12]
ANME-2c-760	ANME-2c	16S rRNA	760-777	50	-	CGC CCC CAG CTT TCG TCC	[12]
ANME-3-1249	ANME-3	16S rRNA	1249-1266	30	-	TCG GAG TAG GGA CCC ATT	[13]
ANME-3-1249H3	Helper	16S rRNA	1249-1266	30	-	GTC CCA ATC ATT GTA GCC GGC	[14]
ANME-3-1249H5	Helper	16S rRNA	1249-1266	30	-	TTA TGA GAT TAC CAT CTC CTT	[14]
Arch915	most *Archaea*	16S rRNA	915-934	35	-	GTG CTC CCC CGC CAA TTC CT	[15]
BMARm-845	Methanotrophic endosymbiont B. sp.	16S rRNA	845-862	30	-	GCT CCG CCA CTA AGC CTA	[16]
DSS658	*Desulfosarcina/ Desulfococcus*	16S rRNA	658-675	50	-	TCC ACT TCC CTC TCC CAT	[17]
EelMS932	ANME-2	16S rRNA	932-949	50	-	AGC TCC ACC CGT TGT AGT	[11]
EPSI682	*Epsilonproteobacteria*	16S rRNA	682-700	20	-	CGG ATT TTA CCC CTA CAC M	[18]
Eub338-I	most *Bacteria*	16S rRNA	338-355	35	-	GCT GCC TCC CGT AGG AGT	[19]
Eub338-II	most *Planctomycetales*	16S rRNA	338-355	35	-	GCA GCC ACC CGT AGG TGT	[20]
Eub338-III	*most Verrucomicrobiales*	16S rRNA	338-355	35	-	GCT GCC ACC CGT AGG TGT	[20]
LEN338 *	*Lentisphaerae*	16S rRNA	338-355	35	-	GCA GCC TCC CGC AGG AGT	This study
Gam42a	*Gammaproteobacteria*	23S rRNA	1027-1043	35	-	GCC TTC CCA CAT CGT TT	[21]
c-Gam42a	Competitor	23S rRNA	1027-1043	35	-	GCC TTC CCA CTT CGT TT	[21]
Gam660	Potential sulfur-oxidizing *Bacteria*	16S rRNA	660-674	35	-	TCC ACT TCC CTC TAC	[22]
Met-I-444	HMMV-type-I-Methanotrophs	16S rRNA	444-461	60	-	CCT GCC TGT TTT CCT CCC	[13]
MPH732	*Methylophaga*	16S rRNA	732-749	40	-	GTA ATG GCC CAG TGA GTC	[13]
MS1414	*Methanosarcina /-coccoides /-lobus*	16S rRNA	1414-1434	50	-	CTC ACC CAT ACC TCA CTC GGG	[23]
hMS1395	MS1414-helper	16S rRNA	1395-1412	50	-	GGT TTG ACG GGC GGT GTG	[24]
MTC-850	*Methylococcales* clade MMG1/MMG2	16S rRNA	850-867	20	-	ACG TTA GCT CCG CCA CTA	This study
MTMC701	most *Methylococcales*	16S rRNA	701-718	40	-	GTG TTC CTT CAG ATC TCT	[11]
c1-MTMC701	Competitor	16S rRNA	701-718	40	-	GTA TTC CTT CAG ATC TCT	This study
c2-MTMC701	Competitor	16S rRNA	701-718	40	-	GTG TTC CTC CAG ATC TCT	This study
NON338	Nonsense probe	-	-	35	-	ACT CCT ACG GGA GGC AGC	[25]
SEEP1a-473	SEEP-SRB-1a	16S rRNA	473-495	30	-	TTC AGT GAT ACC GTC AGT ATC CC	[26]

* the LEN338 probe was combined with the general bacterial probe mix Eub338I_III for a more complete detection of bacterial clades in marine sediments

References

1. Böer SI, Hedtkamp SIC, van Beusekom JEE, Fuhrman JA, Boetius A, et al. (2009) Time- and sediment depth-related variations in bacterial diversity and community structure in subtidal sands. ISME J 3: 780-791.
2. Ramette A (2009) Quantitative Community Fingerprinting Methods for Estimating the Abundance of Operational Taxonomic Units in Natural Microbial Communities. Appl Environ Microbiol 75: 2495-2505.
3. Mueller L, de Brouwer J, Almeida J, Stal L, Xavier J (2006) Analysis of a marine phototrophic biofilm by confocal laser scanning microscopy using the new image quantification software PHLIP. BMC Ecology 6: 1.
4. Daims H, Lücker S, Wagner M (2006) daime, a novel image analysis program for microbial ecology and biofilm research. Environmental Microbiology 8: 200-213.
5. Holmes AJ, Costello A, Lidstrom ME, Murrell JC (1995) Evidence that participate methane monooxygenase and ammonia monooxygenase may be evolutionarily related. FEMS Microbiol Lett 132: 203-208.
6. Massana R, Murray AE, Preston CM, DeLong EF (1997) Vertical distribution and phylogenetic characterization of marine planktonic Archaea in the Santa Barbara Channel. Appl Environ Microbiol 63: 50-56.
7. Muyzer G, de Waal EC, Uitterlinden AG (1993) Profiling of complex microbial populations by denaturing gradient gel electrophoresis analysis of polymerase chain reaction-amplified genes coding for 16S rRNA. Appl Environ Microbiol 59: 695-700.
8. Cardinale M, Brusetti L, Quatrini P, Borin S, Puglia AM, et al. (2004) Comparison of Different Primer Sets for Use in Automated Ribosomal Intergenic Spacer Analysis of Complex Bacterial Communities. Appl Environ Microbiol 70: 6147-6156.
9. Costello AM, Lidstrom ME (1999) Molecular Characterization of Functional and Phylogenetic Genes from Natural Populations of Methanotrophs in Lake Sediments. Appl Environ Microbiol 65: 5066-5074.
10. Lane DJ, Pace B, Olsen GJ, Stahl DA, Sogin ML, et al. (1985) Rapid determination of 16S ribosomal RNA sequences for phylogenetic analyses. PNAS 82: 6955-6959.
11. Boetius A, Ravenschlag K, Schubert CJ, Rickert D, Widdel F, et al. (2000) A marine microbial consortium apparently mediating anaerobic oxidation of methane. Nature 407: 623-626.
12. Knittel K, Lösekann T, Boetius A, Kort R, Amann R (2005) Diversity and Distribution of Methanotrophic Archaea at Cold Seeps. Appl Environ Microbiol 71: 467-479.
13. Niemann H, Lösekann T, de Beer D, Elvert M, Nadalig T, et al. (2006) Novel microbial communities of the Haakon Mosby mud volcano and their role as a methane sink. Nature 443: 854-858.
14. Lösekann T, Knittel K, Nadalig T, Fuchs B, Niemann H, et al. (2007) Diversity and Abundance of Aerobic and Anaerobic Methane Oxidizers at the Haakon Mosby Mud Volcano, Barents Sea. Appl Environ Microbiol 73: 3348-3362.
15. Stahl DA, Amann R (1991) Nucleic acid techniques in bacterial systematics. In: Stackebrandt E, Goodfellow M, editors. Chichester, England: John Wiley & Sons Ltd. pp. 205-248.
16. Duperron S, Bergin C, Zielinski F, Blazejak A, Pernthaler A, et al. (2006) A dual symbiosis shared by two mussel species, Bathymodiolus azoricus and Bathymodiolus puteoserpentis (Bivalvia: Mytilidae), from hydrothermal vents along the northern Mid-Atlantic Ridge. Environ Microbiol 8: 1441-1447.

17. Manz W, Eisenbrecher M, Neu TR, Szewzyk U (1998) Abundance and spatial organization of Gram-negative sulfate-reducing bacteria in activated sludge investigated by in situ probing with specific 16S rRNA targeted oligonucleotides. FEMS Microbiol Ecol 25: 43-61.
18. Moussard H, Corre E, Cambon-Bonavita M-A, Fouquet Y, Jeanthon C (2006) Novel uncultured Epsilonproteobacteria dominate a filamentous sulphur mat from the 13°N hydrothermal vent field, East Pacific Rise. FEMS Microbiol Ecol 58: 449-463.
19. Amann RI, Binder BJ, Olson RJ, Chisholm SW, Devereux R, et al. (1990) Combination of 16S rRNA-targeted oligonucleotide probes with flow cytometry for analyzing mixed microbial populations. Appl Environ Microbiol 56: 1919-1925.
20. Daims H, Brühl A, Amann R, Schleifer KH, Wagner M (1999) The domain-specific probe EUB338 is insufficient for the detection of all Bacteria: development and evaluation of a more comprehensive probe set. Syst Appl Microbiol 22: 434-444.
21. Manz W, Amann R, Ludwig W, Wagner M, Schleifer K-H (1992) Phylogenetic Oligodeoxynucleotide Probes for the Major Subclasses of Proteobacteria: Problems and Solutions. Syst Appl Microbiol 15: 593-600.
22. Ravenschlag K, Sahm K, Amann R (2001) Quantitative Molecular Analysis of the Microbial Community in Marine Arctic Sediments (Svalbard). Appl Environ Microbiol 67: 387-395.
23. Raskin L, Stromley JM, Rittmann BE, Stahl DA (1994) Group-specific 16S rRNA hybridization probes to describe natural communities of methanogens. Appl Environ Microbiol 60: 1232-1240.
24. Crocetti G, Murto M, Björnsson L (2006) An update and optimisation of oligonucleotide probes targeting methanogenic Archaea for use in fluorescence in situ hybridisation (FISH). J Microbiol Meth 65: 194-201.
25. Wallner G, Amann R, Beisker W (1993) Optimizing fluorescent in situ hybridization with rRNA-targeted oligonucleotide probes for flow cytometric identification of microorganisms. Cytometry 14: 136-143.
26. Schreiber L, Holler T, Knittel K, Meyerdierks A, Amann R (2010) Identification of the dominant sulfate-reducing bacterial partner of anaerobic methanotrophs of the ANME-2 clade. Environ Microbiol 12: 2327-2340.
